# Simultaneous imaging of calcium and contraction in the beating heart of zebrafish larvae

**DOI:** 10.7150/thno.64734

**Published:** 2022-01-01

**Authors:** Jussep Salgado-Almario, Manuel Vicente, Yillcer Molina, Antonio Martinez-Sielva, Pierre Vincent, Beatriz Domingo, Juan Llopis

**Affiliations:** 1 Physiology and Cell Dynamics, Centro Regional de Investigaciones Biomédicas (CRIB) and Facultad de Medicina de Albacete, Universidad de Castilla-La Mancha, C/Almansa 14, 02006 Albacete, Spain.; 2 Sorbonne Université, CNRS, Biological Adaptation and Ageing, UMR 8256, F-75005 Paris, France.

**Keywords:** imaging, calcium, contraction, heart, zebrafish

## Abstract

*In vivo* models of cardiac function maintain the complex relationship of cardiomyocytes with other heart cells, as well as the paracrine and mechanoelectrical feedback mechanisms. We aimed at imaging calcium transients simultaneously with heart contraction in zebrafish larvae.

**Methods:** To image calcium in beating hearts, we generated a zebrafish transgenic line expressing the FRET-based ratiometric biosensor Twitch-4. Since emission ratioing canceled out the motion artifacts, we did not use myosin inhibitors or *tnnt2a* morpholinos to uncouple contraction from changes in calcium levels. We wrote an analysis program to automatically calculate kinetic parameters of the calcium transients. In addition, the ventricular diameter was determined in the fluorescence images providing a real-time measurement of contraction correlated with calcium.

**Results:** Expression of Twitch-4 did not affect the force of contraction, the size of the heart nor the heart rate in 3- and 5-days post-fertilization (dpf) larvae. Comparison of 3 and 5 dpf larvae showed that calcium levels and transient amplitude were larger at 5 dpf, but the fractional shortening did not change. To validate the model, we evaluated the effect of drugs with known effects on cardiomyocytes. Calcium levels and the force of contraction decreased by the L-type calcium channel blocker nifedipine, whereas they increased with the activator Bay-K 8644. Caffeine induced bradycardia, markedly decreased ventricular diastolic calcium levels, increased the size of the calcium transients, and caused an escape rhythm in some larvae.

**Conclusions:** The *Tg(myl7:Twitch-4)* line provides a physiological approach to image systolic and diastolic calcium levels in the heart of zebrafish larvae. Since the heart is beating, calcium levels and contraction can be correlated. This line will be a useful tool to address pathophysiological mechanisms in diseases like heart failure and arrhythmia, in cardiotoxicity studies and for drug screening.

## Introduction

In the heart, electrical excitation triggers a transient Ca^2+^ influx (I_Ca,L_) through L-type voltage-dependent calcium channels (LTCC). This influx elicits Ca^2+^ release from the sarcoplasmic reticulum (SR) through ryanodine receptors (RyR). The binding of Ca^2+^ to myofilaments results in the systolic contraction and ejection of blood. The amplitude and duration of the Ca^2+^ transient is highly controlled by channels, transporters, and numerous regulatory proteins, and determines the strength of the contraction and overall cardiac performance 1, 2. Besides, the adrenergic input enhances the heart rate and the response of myofibrils to Ca^2+^. Systemic factors like blood volume and peripheral resistance, affecting preload and afterload, also influence the cardiac output.

Ca^2+^ dysregulation in the heart is involved in the pathogenesis of inherited and acquired arrhythmias 3-5. In heart failure, mishandling of myocyte Ca^2+^ can occur through several mechanisms: decreased Ca^2+^ uptake by SR Ca^2+^ ATPase (SERCA), increased Ca^2+^ leak from the SR and increased activity of the Na^+^/ Ca^2+^ exchanger (NCX1), all reducing SR Ca^2+^ content 6, or lack of synchronicity due to orphaned RyRs 7. A common consequence of these disbalances is a blunted Ca^2+^ transient and systolic dysfunction 6, 7. The effects of Ca^2+^ sensitizers (as opposed to Ca^2+^ mobilizers) as a therapeutic approach to treat congestive heart failure sometimes go beyond their inotropism, acting on other organs with additional benefits for the patients 8. The study of the interrelation of Ca^2+^ levels with force is key to evaluate cardiac contractile dysfunction, reserve capacity and the efficiency of therapies. In turn, relaxation and ventricular filling depend on the rate of Ca^2+^ decay, the sensitivity of myofilaments to Ca^2+^, the passive mechanical properties of the ventricular walls and the atrio-ventricular pressure difference. Accordingly, altered regulation of diastolic and/or resting Ca^2+^ seems to be an important factor involved in heart failure with preserved ejection fraction (diastolic dysfunction) 9, 10. However, little is known about the mechanisms that determine end-diastolic Ca^2+^, as most methods to measure Ca^2+^ rely on the fluorescence change in systole relative to diastole. A second reason is that many studies were performed at artificially low pacing rate, when only sarcolemmal fluxes influence diastolic and resting Ca^2+^ 9.

Given the pivotal role of Ca^2+^ in the heart, numerous studies mostly in isolated cardiomyocytes have provided a detailed insight of the Ca^2+^ handling mechanisms in physiology 1 and disease 10. *In vivo* models do not allow investigating cellular mechanisms with such precision but preserve the interaction between cardiomyocytes and other cell types in the heart, maintaining their paracrine regulation 11. They also retain the link of the heart with the circulation and with other organs and allow measurement of cardiac endpoints such as the ejection fraction, stroke volume and cardiac output. Thus, *in vivo* models of heart function are complementary to studies in the isolated perfused organ and in cells.

Zebrafish embryos, larvae and adults are widely used as *in vivo* animal models in cardiology 12, 13 despite their morphological differences with humans. Studies of development, pathophysiology, drug screening or toxicology are feasible in this small vertebrate, constituting an attractive alternative to mammalian models and facilitating the goal of reduction in their use in research. Heart rate and the ECG, in particular the QT interval, are similar in zebrafish and human 14, 15. The ventricular action potential in zebrafish also possesses a long plateau phase and many channels responsible for the action potential in humans are present in zebrafish, particularly for repolarization 14-18. However, differences in excitation-contraction coupling with the human have also been found 18. In particular, the contribution of Ca^2+^ release from the SR to contraction in adult zebrafish cardiomyocytes is controversial. Since the I_Ca,L_ current density was found to be about 5-fold larger than in pig or human cardiomyocytes 19, the SR contribution to the Ca^2+^ transient is probably less than in mammalians. In another study, only 20% of the action potential-induced Ca^2+^ transient was mediated by release from SR 20. However, a study in heart slices from adult zebrafish found a substantial role (52 to 54%) of SR Ca^2+^ release in force generation 21. To overcome the differences between the fish model and human, steps have been proposed to 'humanize' zebrafish 18, 22.

Many cardiomyopathies and arrhythmias have a genetic base. The zebrafish genome has 70% similarity to human 23 and gene editing techniques can be easily applied 22. Thus, zebrafish is a suitable model to test *in vivo* disease gene candidates and to unveil the link between genotype and phenotype, for instance, in post-GWAS functional studies 24. Several pathophysiological mechanisms elucidated in zebrafish have been confirmed in human induced pluripotent stem cell-derived cardiomyocytes 25. Furthermore, cardiovascular toxicity is an important cause of drug attrition during drug discovery and withdrawal in clinical phases. Zebrafish has been proposed as an *in vivo* model in early drug discovery and in cardiotoxicity studies since it is amenable to high throughput screening 22, 26-28. In one study, zebrafish larvae were found to predict better cardiovascular liabilities in humans than cellular systems 27.

Zebrafish are advantageous to image cardiac function with optical probes, as the heart develops rapidly while the larva is still translucent 29. Thus, several studies have applied non-invasive Ca^2+^ imaging techniques in the heart under physiological conditions and pathological modelling. This approach involves the expression of a genetically encoded Ca^2+^ indicator (GECI), like the single-fluorophore GCaMPs 30, which have been successfully used to image Ca^2+^ dynamics in embryonic hearts 31, 32. Although GCaMP imaging is technically simple, the measurement is highly sensitive to movements. Therefore, it required the use of morpholino oligomers against myosin II to stop heart beating. However, since morpholinos are degraded in cells, this limits imaging to 3- or 4-days post-fertilization (dpf), time after which the heart restarts beating. An alternative is the use of myosin II inhibitors like blebbistatin 33 or its analogs to uncouple contraction from Ca^2+^ transients, an approach not devoid of limitations due to its photosensitivity and degradation. Importantly, the inhibition of contraction prevents the study of the mechanical parameters outlined above and may potentially affect heart physiology and development. We reasoned that a ratiometric Ca^2+^ biosensor, by acquiring two wavelength bands simultaneously, could allow assessment of Ca^2+^ levels in moving hearts, since the ratio is able to cancel out what is common in the two images (motion) while preserving what is different (Ca^2+^) 34-36. Moreover, this approach would allow correlating Ca^2+^ levels with motion and contraction during the cardiac cycle, preserving mechano-electrical feedback 37, normal development and ionic expression.

In an earlier report, we screened several ratiometric Ca^2+^ biosensors with different affinity and kinetic properties to validate their use in the zebrafish larva heart in transient expression experiments 36. Twitch-4 38, an *Opsanus* troponin-C-derived biosensor, provided the largest ratio change during the cardiac cycle with good signal-to-noise. In this work, we have established a transgenic line, *Tg(myl7:Twitch-4)*, to characterize the Ca^2+^ transients and contraction in beating hearts. We validated this model by testing drugs with known effects in the heart at two developmental stages, 3 and 5 dpf.

## Methods

### Zebrafish husbandry

Zebrafish were kept in the Center for Animal Experimentation of the Albacete School of Medicine with a light/dark cycle of 14/10 h. Fertilized zebrafish eggs were obtained following standard procedures and maintained in E3 medium (5 mM NaCl, 0.17 mM KCl, 0.33 mM MgSO_4_, 0.33 mM CaCl_2_, pH 7.4 in double distilled water) at 28.5 ºC. No methylene blue was added to E3 medium to decrease larva autofluorescence. All animal procedures were carried out in compliance with national and EU regulations (approval document dated 16 March 2020, Consejería de Agricultura, Agua y Desarrollo Rural, Junta de Comunidades de Castilla-La Mancha, Spain).

### Generation of transgenic zebrafish lines

The Ca^2+^ biosensor Twitch-4 38 and the Ca^2+^-insensitive FRET construct ECFP-16aa-EYFP 39 were cloned into the pT2A-Tol2-myl7 transposon vector as previously described 36. To generate stable transgenic zebrafish, single-cell wild-type AB embryos were injected with a mixture of pTol2- myl7:Twitch-4 or pTol2-myl7:ECFP-16aa-EYFP cDNA and transposase mRNA, each at a concentration of 12.5 ng/µL. For transposase mRNA production, the pCS-zT2TP construct 40 was linearized via ApaΙ digestion and *in vitro* transcription was carried out using the mMESSAGE mMACHINE SP6 kit (Ambion Inc., TX, USA). Injected embryos (F0) were screened by fluorescence in the heart and grown to adulthood. Adult F0 fish were outcrossed to wild-type AB zebrafish to identify founders with insertions in the germline by the cardiac fluorescence in the F1 offspring. To generate the *Tg(myl7:Twitch-4)* line, fluorescent F1 fish were outcrossed to wild-type AB zebrafish, and F2 embryos expressing Twitch-4 were raised to adulthood and intercrossed to generate the F3 generation. Adult F3 *Tg(myl7:Twitch-4)* fish were crossed with adult translucent *Casper* zebrafish (*roy*^-/-^; *nacre*^-/-^) 41 to obtain F4 embryos. F1 *Tg(myl7:ECFP-16aa-EYFP)* and F4 *Tg(myl7:Twitch-4)* heterozygous larvae were used for imaging.

### Mounting of larvae for microscopy

**Figure 1:**
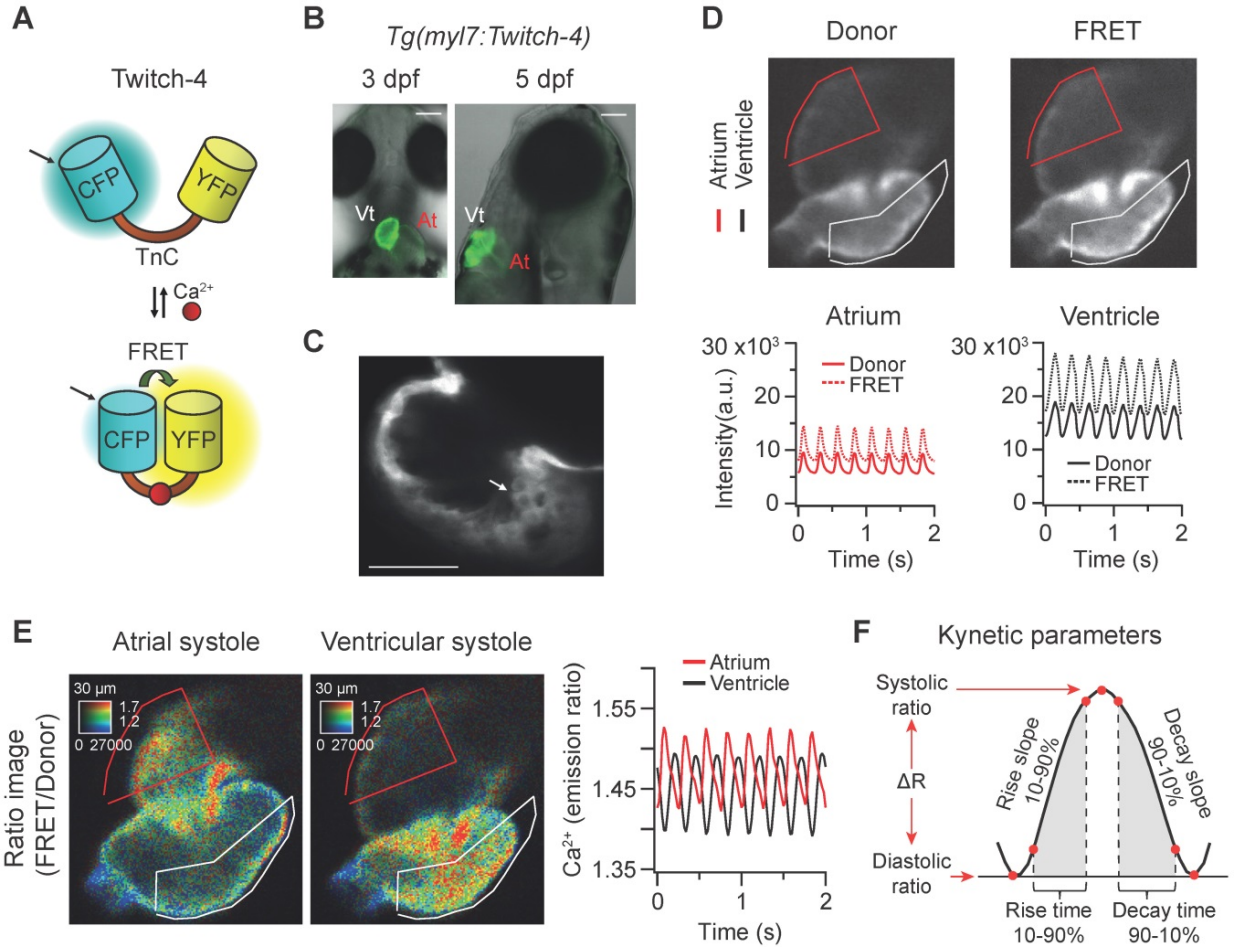
***In vivo* recording of Ca^2+^ dynamics in the beating heart of *Tg(myl7:Twitch-4)* zebrafish larvae. (A)** Schematic structure of the Ca^2+^ biosensor Twitch-4 and its principle of action (FRET, Förster resonance energy transfer). **(B)** Overlay of transmitted light and fluorescence images of 3 and 5 dpf larvae showing the ventral and lateral orientation of the heart after mounting (At - atrium, Vt - ventricle). The scale bar represents 100 μm. **(C)** Confocal image of a 3 dpf larva showing the cytoplasmatic localization of Twitch-4 in the cardiomyocytes. The scale bar represents 50 μm. **(D)** Fluorescence intensity images of the donor and FRET channels of a 3 dpf larva heart in ventricular systole. Regions-of-interest (ROI) were manually drawn on the atrium and ventricle and their mean pixel value was obtained at each timepoint. The traces show the time course of fluorescence intensity of the donor and FRET channels. **(E)** Emission ratio images (FRET image/donor image) in pseudo color of atrial and ventricular systoles in a 3 dpf larva. The calibration squares show the distance in µm, whereas the hue codes for the emission ratio and intensity codes for the fluorescence intensity. The traces show the atrial and ventricular Ca^2+^ levels (emission ratios) over time. **(F)** Kinetic parameters calculated from the ratio time course data (see Methods for their definition).

Non-anesthetized larvae were embedded in 100 µL of 0.3% low melting point agarose in E3 medium, preheated to 42 ºC, and gelled on 96-well plates with square wells and flat clear bottom (ibidi, Germany) (one larva per well). Larvae at 3 dpf were mounted ventral-side down and 5 dpf larvae right-side down (Figure 1B) to visualize better atrium and ventricle at these two stages. Once the agarose solidified, 100 µL of E3 medium at 28 ºC was added. After mounting, the larvae were incubated on the microscope stage for 30 min at 28 ºC to get a stable heart rate (HR). Where indicated, larvae were treated with 75 µM of the myosin inhibitor para-amino blebbistatin (Optopharma, Hungary) for 2 h before mounting for microscopy.

### Ratiometric fluorescence imaging

We acquired fluorescence images of the heart of 3 dpf *Tg(myl7:ECFP-16aa-EYFP)* larvae, and 3 and 5 dpf *Tg(myl7:Twitch-4)* larvae with a wide-field fluorescence microscope (DMIRE-2, Leica Microsystems, Germany) equipped with a sCMOS camera (2048 × 2048 pixels, ORCA-Flash 4.0, Hamamatsu Photonics, Japan), controlled by the software Aquacosmos 2.6 (Hamamatsu Photonics, Japan). The image acquisition rate was 50 Hz (20 ms integration per image) during 5-10 s; some larvae were imaged at 100 Hz. Larvae under the microscope stage were kept at 28ºC in a chamber incubator (PeCon GmbH, Germany). To excite the fluorophores, light from a LED source (Lambda TLED+, Sutter Instrument, CA, USA) was applied continuously for 5-10 s using a 440AF21 nm bandpass filter (Chroma, VT, USA) and a beamsplitter 455DLRP (Omega Optical, VT, USA). A 10x air objective (HC PlanApo 0.45 NA, Leica Microsystems, Germany) was used. The donor (CFP) and acceptor (FRET) images were acquired simultaneously with an image splitter (W-View Gemini, Hamamatsu Photonics, Japan), which divided the camera field in two halves corresponding to donor and acceptor emission. Fluorescence emission was separated with beamsplitter (509-FDi01, Semrock, NY, USA) and passed through 483/32 nm and 542/27 nm emission filters (Semrock, NY, USA). Images were acquired in 16 bits with 2 × 2 binning. With this configuration, the resolution of the images was 1.45 µm × 1.45 µm/pixel. The FRET image corresponds to the cpCitrine174 or EYFP emission (542/27 nm) at the donor excitation (440AF21 nm), and the donor image corresponds to the ECFP emission (483/32 nm) at the donor excitation (440AF21 nm).

### Drug treatment

For drug response experiments, stock solutions of nifedipine (Sigma-Aldrich N7634), (±)-Bay K8644 (Tocris 1544) and ryanodine (Tocris 1329) were made in dimethyl sulfoxide (DMSO) at concentrations of 10, 20 and 25 mM, respectively. A stock solution of caffeine (Sigma-Aldrich N7634) was made in water at a concentration of 50 mM. Drug stocks were diluted in E3 medium at 28 ºC. After recording the basal images, 100 µL of the drugs were added to the wells, reaching a final concentration of 100 µM for nifedipine, (±)-Bay K 8644 and ryanodine, and 3 mM for caffeine. New sets of images were recorded after the incubation with the drugs, as indicated.

### Image processing and data analysis

Ratiometric images and ratio data were processed and analyzed with an analysis program (Ratioscope) written in the IGOR Pro environment (WaveMetrics, OR, USA). We corrected for pixel shift between FRET and donor images. The ratio *FRET image*/*donor image* was calculated pixel-by-pixel for each time point. Regions of interest (ROI) were drawn over the atrium and the ventricle walls in diastole. The ratio value for a ROI was calculated by averaging all the pixels' values weighted by the average intensity of donor and FRET channels 34, 35. Since the ratio for pixels close to background can reach infinite values, pixels with values smaller than the minimum displayed ratio/4 or larger than the maximum displayed ratio×4 were clipped. If necessary, the Savitzky-Golay smoothing filter was applied to reduce the noise in the raw ratio traces. Several kinetic parameters were automatically calculated from the ratio traces: diastolic ratio (the lowest ratio in the cardiac cycle), systolic ratio (the highest ratio in the cardiac cycle), ratio amplitude (ΔR, systolic minus diastolic ratio), heart rate (HR, in bpm), rise time (time from 10 to 90% of systolic Ca^2+^ rise), decay time (time from 90 to 10% of diastolic Ca^2+^ decay), rise slope (ΔR from 10 to 90% value, divided by the rise time) and decay slope (ΔR from 90 to 10% value, divided by the decay time) (Figure 1F). The Ca^2+^ transients and the points of interest indicated in Figure 1F were detected automatically using the edge detection functions provided in the Igor Pro (WaveMetrics) environment and implemented in a custom-made analysis module (“Pulse” module in the “Ratioscope” package) (Figure S1). Data shown for each larva represent the average of all the cardiac cycles in 5 or 10 s of continuous recording. To monitor contractile parameters in *Tg(myl7:Twitch-4)*, we wrote an analysis program in IGOR Pro that automatically detects contraction events (Figure S1) and records changes in the ventricular diameter during the cardiac cycle (Figure 4A). A line drawn through the ventricle in the fluorescence images tracks the movement of the wall and the displacement of the ventricular walls is represented in a kymogram. The FS was calculated as the difference between the end-diastolic and end-systolic diameters, divided by the end-diastolic diameter. The external wall was used to measure the ventricular diameter since the inner wall did not provide sufficient contrast. The program used to calculate all these measurements in the Igor Pro environment is publicly available as “Ratioscope 8.31” on a data repository site at the link: https://doi.org/10.25493/5G5V-HBC.

Transmitted light images were analyzed manually in ImageJ 42 as previously described 36 to calculate FS, fractional area change (FAC) and HR, where indicated. The FAC was calculated as the difference between the end-diastolic and end-systolic areas, divided by the end-diastolic area.

### GCaMP fluorescence imaging

*Tg(myl7:GCaMP)^s878^* adult zebrafish were outcrossed to wild-type strain and fertilized eggs at 1-cell stage were injected with 2 ng of the morpholino oligomer *tnnt2a* (5'-CATGTTTGCTCTGATCTGACACGCA-3'). Embryos from 24 hours post-fertilization (hpf) were maintained in 0.003% N-phenylthiourea to prevent pigmentation. Larvae were embedded in 1% low melting point agarose and transferred to an 8-well glass bottom plate (ibidi, Germany). Fluorescence images were acquired at a rate of 200 Hz with a CSU X1 spinning disc confocal microscope (Carl Zeiss, Germany) equipped with a Hamamatsu ORCA Flash4.0 sCMOS camera (Hamamatsu Photonics, Japan) in 16 bits with 2 x 2 binning. For image analysis, ROIs were drawn in the atrium and in the ventricle to obtain mean intensity values. An exponentially weighted moving average smoothing with a smoothing factor of 0.7 was applied and data was transformed into ΔF/F_0_ = (F_t_ - F_0_)/F_0_; where F_t_ is the fluorescence at a given time and F_0_ is the minimum diastolic fluorescence value. For characterization of the Ca^2+^ transients, ΔF/F_0_ data were analyzed with Clampfit 10.7 (Molecular Devices, CA, USA) to determine rise time 10% to 90% and decay time 90% to 10%.

### Cardioluminescence

The zebrafish line *Tg(myl7:GFP-Aequorin)* was used to compare averaged Ca^2+^ in 3 and 5 dpf larvae with a luminescence-based method described in Vicente et al 43.

### Confocal microscopy

*Tg(myl7:Twitch-4)* larvae at 3 dpf were euthanized by incubation in 0.3% MS-222 (Sigma-Aldrich A5040) for 5 min. Larvae were embedded in agarose as done for ratiometric imaging and were imaged in an inverted Axio Observer LSM710 confocal microscope (Carl Zeiss, Germany) with a PlanApo 20x/0.8 NA objective. Laser excitation was at 488 nm and the emission bandwidth was 520-560 nm.

### Statistics

Statistical analysis was done with GraphPad Prism 8 (Graphpad Software, CA, USA) and Igor Pro (WaveMetrics, OR, USA). The number of independent experiments (experimental days, N) and the number of larvae (n) are indicated in each figure. The Shapiro-Wilk test was used to test for normality. Differences between two groups were analyzed using the unpaired Student's t-test for parametric data or the Mann-Whitney test for non-parametric data, as indicated. Comparisons between experimental groups were analyzed by one-way repeated measures ANOVA with Dunnett's multiple comparisons post-test for parametric data or the Friedman test with Dunn's multiple comparisons post-test for non-parametric data, as indicated. Data are shown as the mean ± SD. A p < 0.05 was considered statistically significant and significances are indicated as * for p < 0.05, ** p < 0.01, *** p < 0.001, **** p < 0.0001, ns p > 0.05. The Supplementary Data 1 file shows the details of the statistical analysis used throughout.

## Results

### Generation of a transgenic line expressing Twitch-4 in the zebrafish heart

The ratiometric Ca^2+^ biosensor Twitch-4 belongs to a family of GECIs based on the C-terminal domain of the toadfish *Opsanus tau* troponin C as the Ca^2+^-binding domain, with a *Kd* of 2.8 µM and a decay time constant of 0.5 s determined *in vitro* 38. It contains the fluorescent proteins ECFP as the FRET donor at the N-terminal end, and cpCitrine174, a YFP, as the FRET acceptor at the C-terminal end (Figure 1A). We generated a transgenic zebrafish line expressing Twitch-4 under the control of the *myl7* promoter,* Tg(myl7:Twitch-4)*. This promoter drives specific expression in myocardial cells of the atrium, ventricle, and the atrioventricular canal, excluding endocardial and epicardial cells 44. Fish expressing Twitch-4 developed normally and embryos showed specific heart fluorescence starting at 24 hpf. The ventricle was more fluorescent than the atrium due to its thicker wall (Figure 1B). Larvae were mounted ventral-side down (3 dpf) or right-side down (5 dpf) to see atrium and ventricle in the same plane. Confocal microscopy images of 3 dpf *Tg(myl7:Twitch-4)* larvae confirmed the expected cytoplasmic localization of the biosensor (Figure 1C).

Chemical Ca^2+^ indicators and GECIs can act as exogenous buffers and potentially alter the Ca^2+^ dynamics and downstream events 45. Therefore, we determined whether the expression of Twitch-4 affected some functional parameters in the heart of zebrafish larvae. The ventricular fractional shortening (FS) and fractional area change (FAC) represent the relative change of diameter and ventricular area during systole (see Methods for their definition). The FS and FAC of 3 and 5 dpf *Tg(myl7:Twitch-4)* larvae, measured by transmitted light, were similar to those of their non-expressing siblings (Figure S2A). Moreover, we found no differences in the ventricular end-diastolic and end-systolic areas (Figure S2B), as well as in the HR (Figure S2C). Thus, the expression of Twitch-4 in the embryonic zebrafish heart did not appear to affect the cardiac contractility, the size of the heart nor the HR.

### Ratiometric Ca^2+^ imaging in the beating heart of zebrafish larvae

To study Ca^2+^ kinetics in the moving heart, we performed ratiometric imaging of 3 dpf *Tg(myl7:Twitch-4)* larvae acquiring simultaneously the fluorescence of the donor ECFP and FRET channels (both with donor excitation; see channel definition in Methods). The fluorescence intensity in both emission channels showed fluctuations, which were positively correlated, increasing during systole, and decreasing during diastole (Figure 1D), due to the movement of the heart. The FRET channel had two components that increased fluorescence in systole: the movement and the increased Ca^2+^ (high FRET); in the donor channel the heart movement increased the fluorescence, but high Ca^2+^ (high FRET) decreased it. Therefore, the relative fluorescence change was larger in the FRET channel than in the donor channel. Ratioing these channels (FRET/donor) cancelled out the effect of motion and augmented the effect of the Ca^2+^ rise (the reciprocal component). The ratiometric images and the ratio traces were generated with an intensity weighted method. Thus, pixel average ratios were computed without the need to set intensity thresholds 34-36 (Figure 1E). The emission ratio values in atrium and ventricle oscillated synchronously with the heart contractions (Figure 1E). Several parameters were calculated from the ratio traces to characterize the kinetics of the Ca^2+^ transients: diastolic and systolic ratio, amplitude (∆R), as well as rise and decay times and slopes (Figure 1F; see Methods).

### Ratiometric imaging with Twitch-4 corrects motion artifacts

**Figure 2:**
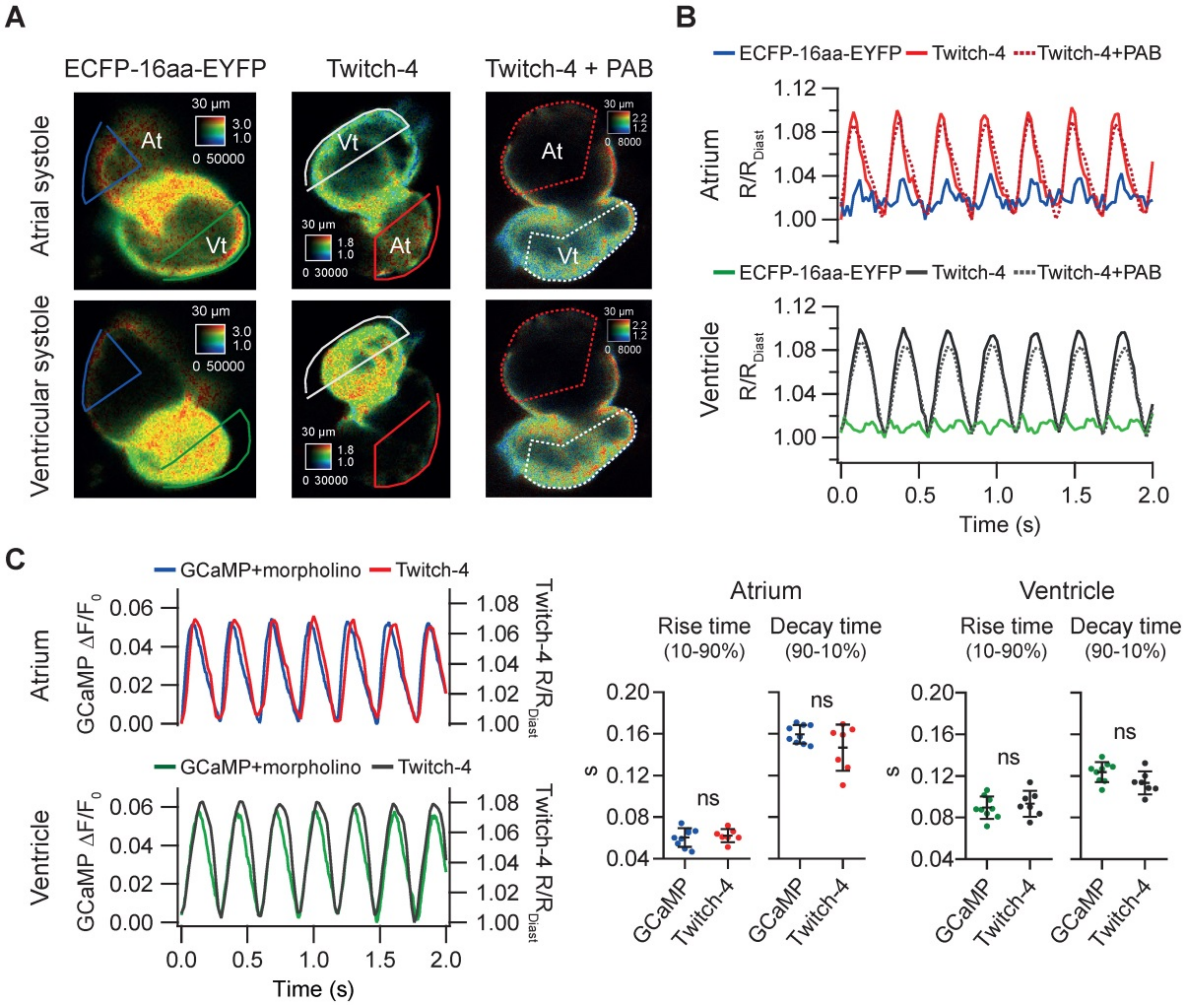
***Tg(myl7:Twitch-4)* reports cardiac Ca^2+^ oscillations and corrects motion artifacts in 3 dpf larvae. (A)** Emission ratio images of *Tg(myl7:ECFP-16aa-EYFP)* and *Tg(myl7:Twitch-4)* moving hearts, and *Tg(myl7:Twitch-4)* heart stopped with 75 µM para-amino blebbistatin (PAB) (representative experiments). The atrial (At) and ventricular (Vt) systoles are shown. **(B)** Normalized emission ratio traces over time (R/R_Diast_) calculated from larvae in A. The lowest diastolic ratio value (R_Diast_) in each register was used for normalization. **(C)** Representative atrial and ventricular Ca^2+^ transients of a *Tg(myl7:GCaMP)^s878^* non-contracting heart (*tnnt2a* mopholinos) and a *Tg(myl7:Twitch-4)* beating heart. The plots show the rise and decay times of the atrial and ventricular Ca^2+^ transients (n = 9 larvae for *Tg(myl7:GCaMP)^s878^* from N = 2 experiments, and n = 7 for *Tg(myl7:Twitch-4)* from N = 5 experiments). Data are shown as the mean ± SD. A two-tailed unpaired Student's t-test was used.

We wanted to determine whether the change in ratio observed in *Tg(myl7:Twitch-4)* larvae was indeed reporting cardiac Ca^2+^ fluctuations and to what extent it was affected by motion of the heart. We generated a zebrafish transgenic line that expresses in the heart a FRET construct with no Ca^2+^-binding domain, composed of an ECFP and an EYFP joined by a flexible linker of 16 amino acid residues 39. In *Tg(myl7:ECFP-16aa-EYFP)* all the fluorescence fluctuations must be entirely due to motion and therefore this artifact should be cancelled in the emission ratio. We observed a small periodic ratio change in *Tg(myl7:ECFP-16aa-EYFP)* compared to *Tg(myl7:Twitch-4)* (Figure 2A-B). It was due to autofluorescence of the yolk under the heart (Figure S3) and it affected mostly the atrium since it was dimmer than the ventricle. We compared the variance of the emission ratio of both constructs to estimate the contribution of motion to the recorded ratios. In 3 dpf larvae the emission ratio variance of ECFP-16aa-EYFP was 18% and 2.3% of the variance of Twitch-4 in the atrium and ventricle, respectively (n = 9 larvae, N = 2 experiments for ECFP-16aa-EYFP; n = 9, N = 4 for Twitch-4). Thus, motion affected the atrium but hardly influenced Twitch-4 emission ratios in the ventricle, where the specific Twitch-4 fluorescence was dominant and autofluorescence was negligible. The effect of motion should be reduced in 5 dpf embryos since the yolk sac is smaller and does not overlap with the heart. As yolk autofluorescence was heterogeneous in space (Figure S3) it was not subtracted in Twitch-4 imaging. In support of the above contention, there was no difference in Twitch-4 ratio changes between moving hearts and hearts stopped with the myosin inhibitor para-amino blebbistatin (Figure 2A-B). The kinetics of Twitch-4 ratio changes in moving hearts were also compared to those obtained with the intensiometric GECI GCaMP in hearts stopped with *tnnt2a* morpholinos (Figure 2C). In larvae with matching HR, the rise time and decay time in atrium and ventricle were similar between both biosensors, as was the shape of the Ca^2+^ transients. In conclusion, the ratiometric measurements with Twitch-4 largely corrected the motion artifacts and reflected the variations in Ca^2+^ levels in the beating heart.

### Characterization of cardiac Ca^2+^ kinetics in 3 and 5 dpf zebrafish larvae

**Figure 3:**
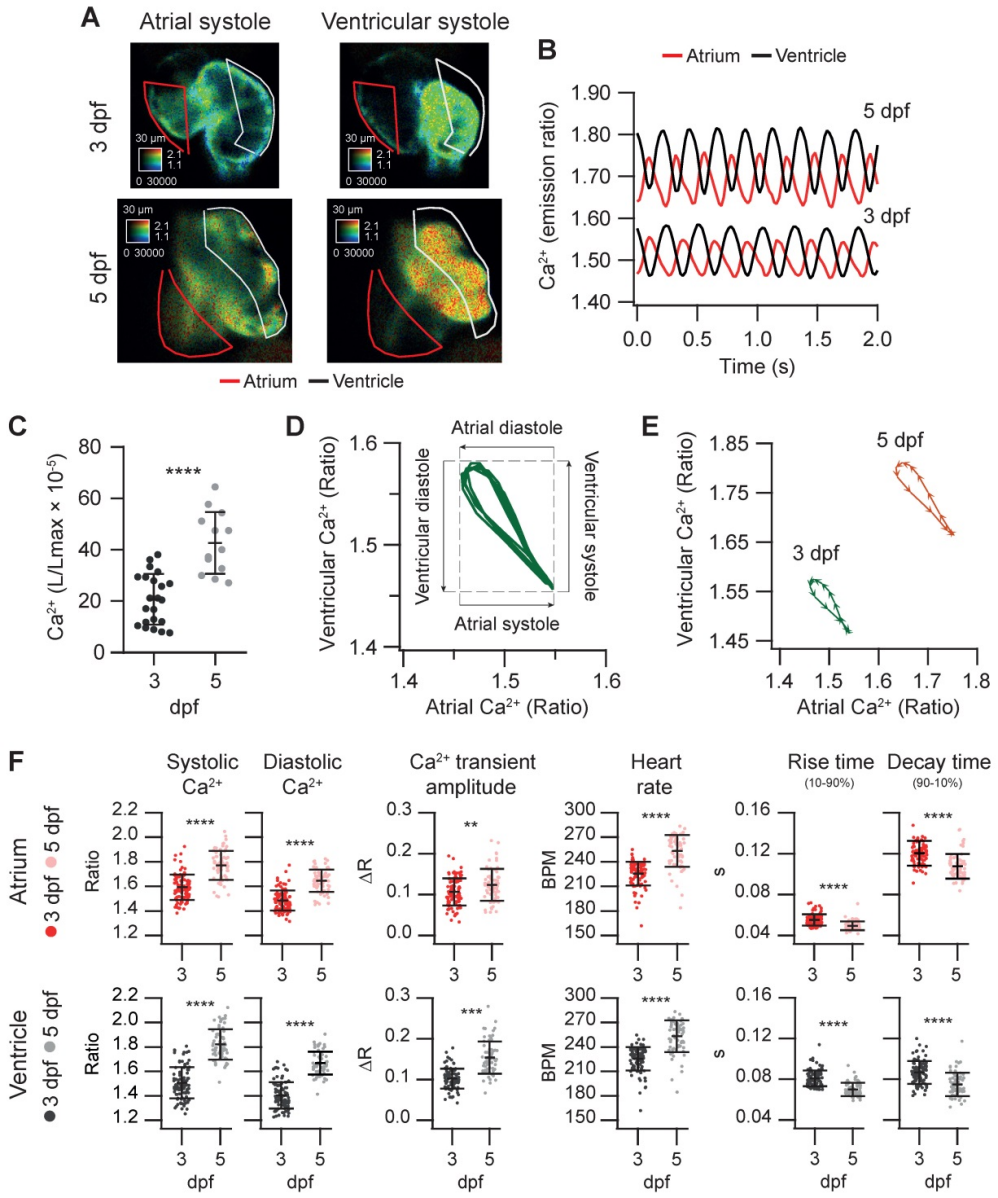
**Basal cardiac Ca^2+^ kinetics of 3 and 5 dpf zebrafish larvae. (A)** Emission ratio images of atrial and ventricular systoles of 3 and 5 dpf *Tg(myl7:Twitch-4)* representative larvae. **(B)** Atrial and ventricular Ca^2+^ levels (emission ratio) over time calculated from larvae in A. **(C)** Ventricular Ca^2+^ levels (L/Lmax) of 3 (n = 22, N = 8) and 5 dpf (n = 13, N = 5) *Tg(myl7:GFP-Aequorin)* larvae measured with a luminescence method. **(D)** Lissajous diagrams of the ventricular *vs.* atrial Ca^2+^ in several cardiac cycles from the 3 dpf larva traces in B. The arrows indicate the phases of the cardiac cycle. **(E)** Diagrams of the ventricular *vs.* atrial Ca^2+^ levels of the 3 and 5 dpf larvae in B (one cardiac cycle). The distance between arrowheads in these loops represents 20 ms, showing the direction of time and the relative speed of each phase. **(F)** Kinetic parameters extracted from atrial and ventricular Ca^2+^ traces of 3 (n = 100, N = 12) and 5 dpf (n =68, N = 7) *Tg(myl7:Twitch-4)* larvae (see Methods for parameter definition). Statistical analysis was performed as indicated in Supplementary data 1. Data are shown as mean ± SD (*** p < 0.001, **** p < 0.0001).

We used the *Tg(myl7:Twitch-4)* line to characterize cardiac Ca^2+^ kinetics at two stages of development, 3 and 5 dpf. The ratio levels and amplitude were higher at 5 dpf than at 3 dpf (Figure 3A and B and Movie S1), suggesting differences in the cardiac Ca^2+^ levels. We attempted to calibrate the emission ratios in terms of intracellular free Ca^2+^ concentration by incubating the larvae with ionomycin in high Ca^2+^ in the E3 medium, or zero Ca^2+^ with EGTA, but the results were not conclusive, probably because the ionophore did not fully reach the cardiomyocyte membrane. Nevertheless, as Twitch-4 emission ratio is proportional to Ca^2+^ levels, our results suggest an increase in the cardiac Ca^2+^ levels at 5 dpf. This was confirmed with an independent method using GFP-Aequorin, a bioluminescent Ca^2+^ indicator, in a *Tg(myl7:GA)* zebrafish line that we have generated 43. The L/Lmax value, which is proportional to Ca^2+^ levels, was higher in the ventricle of 5 dpf larvae than in 3 dpf larvae (Figure 3C).

The electrical excitation starts in the sinoatrial node and is propagated to the ventricle with a delay to allow atrium and ventricle to contract and relax alternately, allowing ventricular filling. This asynchrony was observed in atrial and ventricular Ca^2+^ changes (Figure 3B and Movie S1). We correlated ventricular and atrial Ca^2+^ using Lissajous diagrams (Figure 3D). When atrial Ca^2+^ levels increased, ventricular Ca^2+^ levels decreased and vice versa. After ventricular Ca^2+^ reached a peak, the levels in both chambers decreased simultaneously for a brief time, showing a hysteresis in the loop. Figure 3E shows these loops for 3 and 5 dpf larvae in one cardiac cycle. The arrowheads were spaced 20 ms (each represents one image), showing the direction of time and the relative speed of each phase. Regarding the kinetic parameters of the Ca^2+^ transients, at 5 dpf the amplitude was larger, the HR was faster, and rise and decay times were shorter (Figure 3E-F). At both developmental stages atrial rise time was shorter than the decay time, whereas in the ventricle they were comparable, in agreement with previous reports 31, 36.

### Simultaneous measurement of cardiac Ca^2+^ levels and contractile function

**Figure 4:**
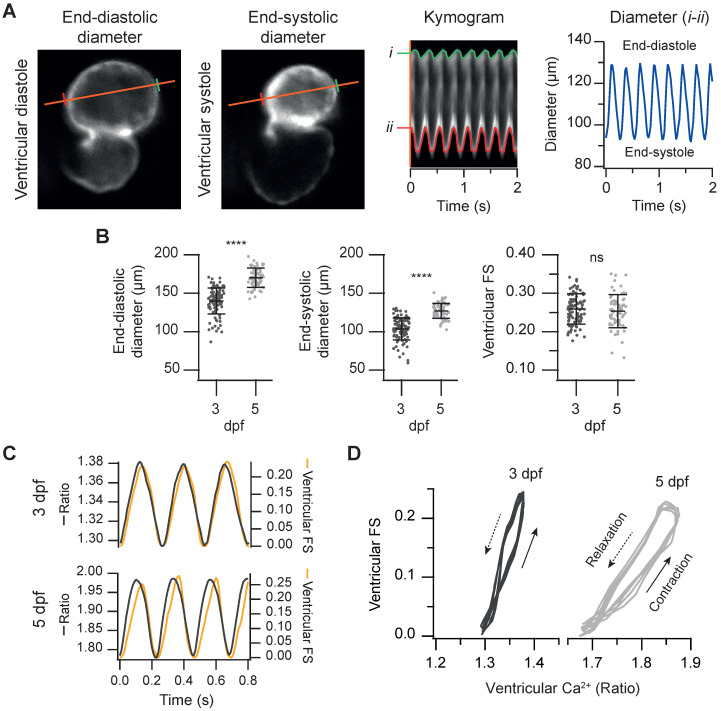
** Simultaneous measurement of the ventricular Ca^2+^ levels and contractile function in 3 and 5 dpf zebrafish larvae. (A)** FRET channel images of a representative 3 dpf *Tg(myl7:Twitch-4)* larva showing end-systolic and end-diastolic ventricular diameters. The kymogram displays the changes of diameter along time (the distance between red and green lines). The trace shows the ventricular diameter extracted from the kymogram. **(B)** Ventricular end-diastolic and end-systolic diameters, and the ventricular FS of 3 (n = 100, N = 12) and 5 dpf (n = 68, N = 7) *Tg(myl7:Twitch-4)* larvae. Data are shown as mean ± SD. Statistical analysis was performed as indicated in Supplementary data 1 (**** p < 0.0001). **(C)** FS and ventricular Ca^2+^ changes of representative 3 and 5 dpf *Tg(myl7:Twitch-4)* larvae over time. **(D)** Diagrams of the ventricular FS *vs*. ventricular Ca^2+^ level of the larvae in C during several cardiac cycles. The arrows indicate the direction of time.

Since ratiometric imaging of *Tg(myl7:Twitch-4)* was performed on moving hearts, we measured simultaneously ventricular Ca^2+^, the ventricular diameter and the FS, as a proxy of the contractility. The same FRET channel images used to measure Ca^2+^ were used to monitor continuously changes in ventricle shape. To this end, we wrote an analysis program that automatically tracks the ventricular wall in the fluorescence images to construct kymograms and obtain the diameter over time (see Methods). Thus, we obtained a continuous recording of ventricle diameter (Figure S1) so that FS could be calculated continuously (Figure 4A and C). As expected, the larvae displayed larger end-diastolic and end-systolic diameters at 5 dpf than at 3dpf, but no statistical differences were found in the FS (25.9 ± 3.9% for 3 dpf and 25.3 ± 4.3% for 5 dpf, Figure 4B). Plotting together Ca^2+^ and FS over time showed that the Ca^2+^ rise was followed by the contraction with a short delay (Figure 4C). The interval between the systolic Ca^2+^ peak and maximal FS was significantly longer at 5 dpf than at 3 dpf (44 ± 15 ms at 5 dpf *vs*. 17 ± 6 ms at 3 dpf, average ± SD, p < 0.0001 in images acquired at 100 Hz). However, there is some uncertainty in these values due to the kinetics of Twitch-4 (see Discussion). Plotting the FS *vs.* Ca^2+^ (Ca^2+^-contraction curves) showed a loop with a contraction limb and a relaxation limb (Figure 4D). The delay between the Ca^2+^ rise and heart shortening resulted in a larger hysteresis of these loops at 5 dpf. In addition, the slope of the contraction-Ca^2+^ loop was lower at 5 dpf suggesting that Ca^2+^ levels changed more to obtain the same FS. These results illustrate a further advantage of Twitch-4 compared to intensiometric Ca^2+^ biosensors: the ability to correlate Ca^2+^ changes and the heart's mechanical function in real time.

### Effect of an LTCC blocker and an LTCC activator on cardiac Ca^2+^ and ventricular shortening in zebrafish larvae

**Figure 5:**
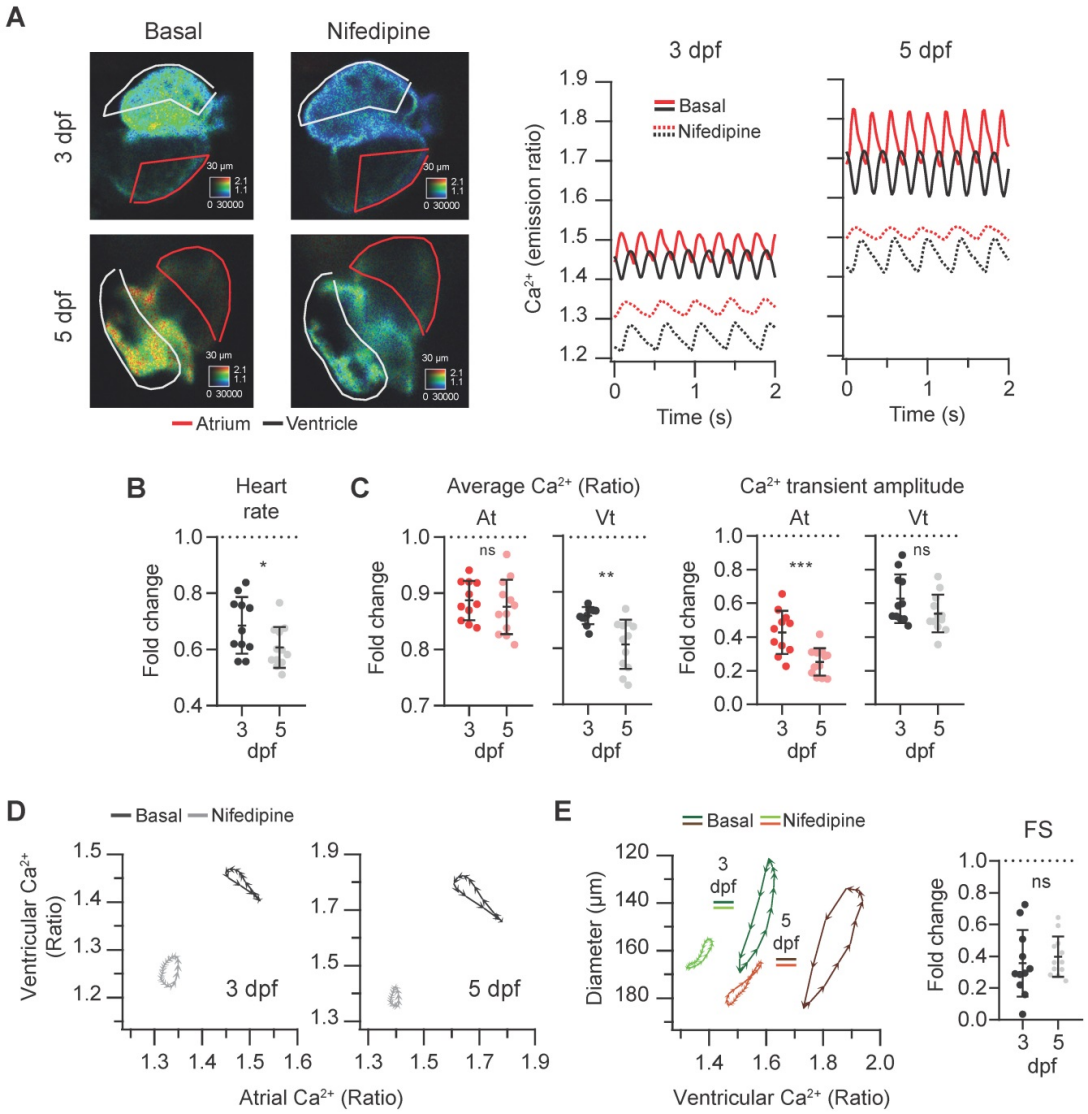
** Effect of the L-type Ca^2+^ channel blocker nifedipine on cardiac Ca^2+^ levels and ventricular shortening of 3 and 5 dpf zebrafish larvae.** *Tg(myl7:Twitch-4)* larvae at 3 (n = 11, N = 4) and 5 dpf (n = 12, N = 4) were treated with 100 µM nifedipine for 1 h. **(A)** Emission ratio images of a ventricular systole of representative larvae before (basal) and after the incubation with nifedipine. The traces show the atrial (red) and ventricular (black) Ca^2+^ levels (emission ratio) of these larvae. **(B)** Fold change over the basal HR of larvae treated with nifedipine. **(C)** Fold change of the average Ca^2+^ levels and amplitude of Ca^2+^ transients over their basal values in the atrium (At) and ventricle (Vt) **(D)** Diagrams of the ventricular *vs*. atrial Ca^2+^ levels (one cardiac cycle) of representative larvae before (basal) and after the incubation with nifedipine (note the different scale for 3 and 5 dpf). **(E)** Diagrams of the ventricular diameter *vs*. ventricular Ca^2+^ level (one cardiac cycle) of representative larvae before (basal) and after addition of nifedipine. The plot shows the fold change over the basal FS. All data are shown as the mean ± SD. A two-tailed unpaired Student's t-test was used (* p < 0.05, ** p < 0.01, *** p < 0.001).

To assess the sensitivity of the *Tg(myl7:Twitch-4)* line to detect changes in Ca^2+^ levels we tested the effects of a blocker and an activator of LTCC on Ca^2+^ kinetics and heart contractility. Since drugs added to the bath have to diffuse through the agarose layer and the skin to reach the internal medium, nifedipine was tested at 1, 10 and 100 µM. It decreased the HR and Ca^2+^ levels at all doses and we compared the effect of 100 µM nifedipine at 3 and 5 dpf. The atrial and ventricular Ca^2+^ levels and the HR decreased, particularly at 5 dpf, and the amplitude of the Ca^2+^ transients decreased more in atrium than in ventricle (Figure 5A-C and Movie S2). The altered Ca^2+^ kinetics changed the shape of the loop in the ventricular *vs.* atrial Ca^2+^ plots (Figure 5D). In addition, the end-systolic diameters increased (less shortening) concomitantly with the reduced Ca^2+^ levels (Figure 5E). Thus, nifedipine markedly reduced the contractility: the FS was 35% and 40% of its basal value at 3 and 5 dpf, respectively (Figure 5E).

**Figure 6:**
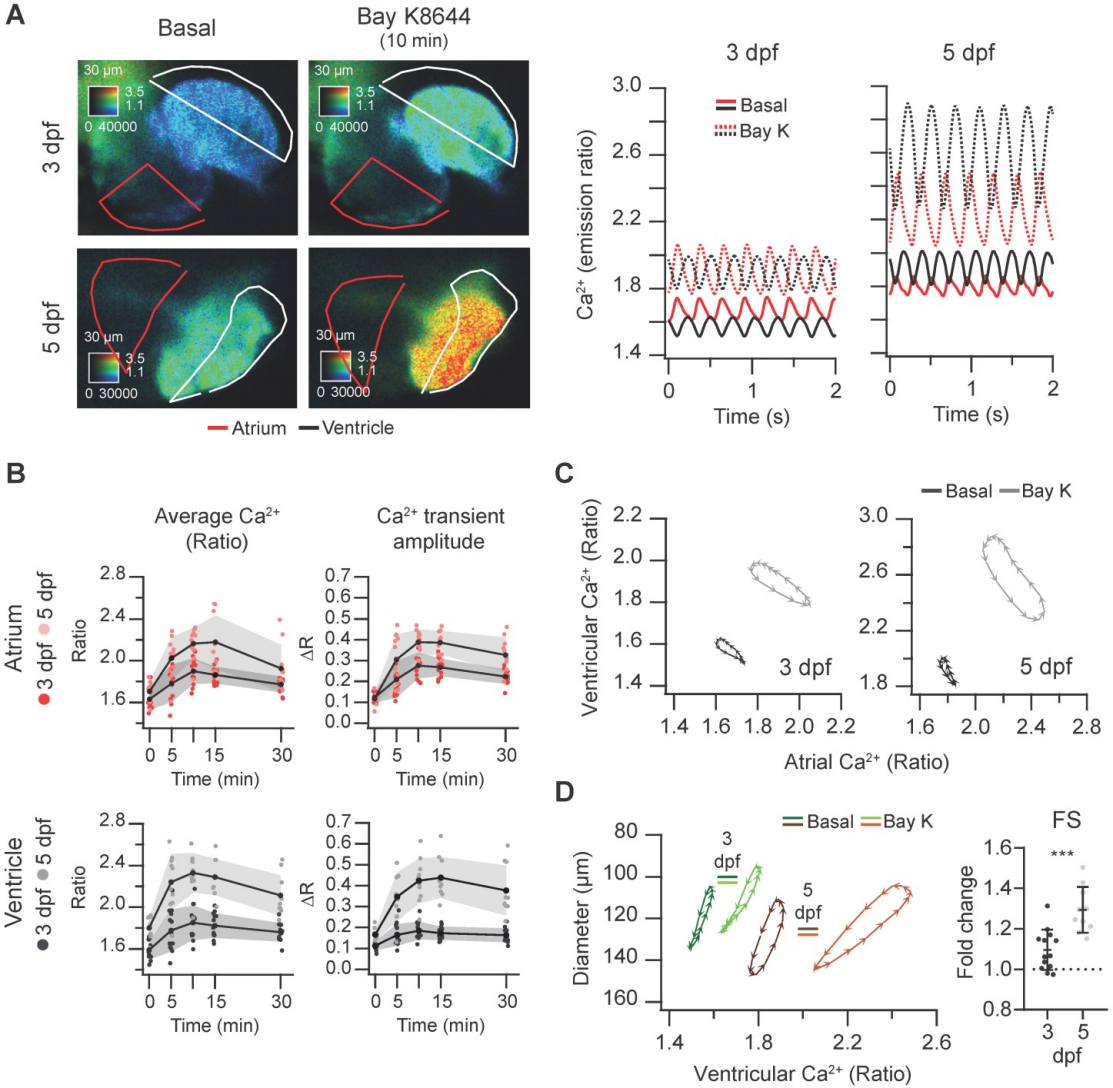
** Effect of the L-type Ca^2+^ channel activator Bay K8644 on cardiac Ca^2+^ levels and ventricular shortening of 3 and 5 dpf zebrafish larvae.** *Tg(myl7:Twitch-4)* larvae at 3 (n = 13, N = 3) and 5 dpf (n = 10, N = 3) were treated with 100 µM Bay K8644. **(A)** Emission ratio images of a ventricular systole of representative larvae before (basal) and after 10 min incubation with Bay K8644. The traces show their atrial (red) and ventricular (black) Ca^2+^ levels (emission ratio). **(B)** Effect of Bay K8644 on the average Ca^2+^ levels and Ca^2+^ transient amplitude in the atrium and ventricle. Data are shown as the mean (black line) ± SD (gray stripe). **(C)** Diagrams of the ventricular *vs*. atrial Ca^2+^ levels (one cardiac cycle) of representative larvae before (basal) and after 10 min incubation with Bay K8644. **(D)** Diagrams of the ventricular diameter *vs*. ventricular Ca^2+^ level (one cardiac cycle) of representative larvae before (basal) and after 10 min incubation with Bay K8644. The plot shows the fold change over the basal FS of larvae treated with Bay K8644 for 10 min. A two-tailed unpaired Student's t-test was used (*** p < 0.001).

In contrast with nifedipine, the LTCC activator Bay K8644 at 100 µM markedly increased the Ca^2+^ levels of 3 and 5 dpf *Tg(myl7:Twitch-4)* larvae (Figure 6A and Movie S3), reaching the maximum effect after 10 min (Figure 6B). These changes were more pronounced at 5 dpf in both chambers (Figure S4A). The amplitude and hysteresis of ventricular *vs*. atrial Ca^2+^ loops increased (Figure 6C). The Ca^2+^-contraction curves of 3 and 5 dpf larvae showed shorter end-systolic diameters, in line with the rise in Ca^2+^ levels (Figure 6D and S4B). However, the end-diastolic diameter decreased 6% at 3 dpf but not at 5 dpf (Figure S4B), and the FS increased more at 5 dpf (Figure 6D). In zebrafish atrial contraction is the main determinant of ventricular filling, rather than passive filling from the veins. Since Bay K8644 also increased atrial Ca^2+^ levels (Figure 6A), stronger atrial contraction may contribute to larger FS in 5 dpf larvae. In summary, Bay K8644 increased Ca^2+^ transient amplitude, heart shortening and contractility.

### Effect of ryanodine and caffeine on cardiac Ca^2+^ levels and ventricular shortening of zebrafish larvae

We examined the effects of compounds acting on RyR on Ca^2+^ levels and heart shortening. First, we tested the response of 3 and 5 dpf *Tg(myl7:Twitch-4)* larvae to the inhibition of RyR by incubation with 1, 10 and 100 µM ryanodine. Effects were observed only with 100 µM and were maximal at 2 h. The amplitude of the Ca^2+^ transients decreased in the ventricle of 3 and 5 dpf larvae compared to larvae treated with DMSO for 2 h (Figure S5A-C) but the FS did not change significantly (Figure S5D). Ryanodine slightly decreased Ca^2+^ levels with no difference in the ventricular diameters (Figure S5A and E and Supplementary data 1). It is possible that the relatively small effects found with ryanodine are due to the reported minor contribution of the SR to the Ca^2+^ transient in zebrafish, compared to mammals 19, 20. However, since we do not know the degree of ryanodine receptor inhibition in our experiments, no conclusion can be drawn regarding this controversy.

**Figure 7:**
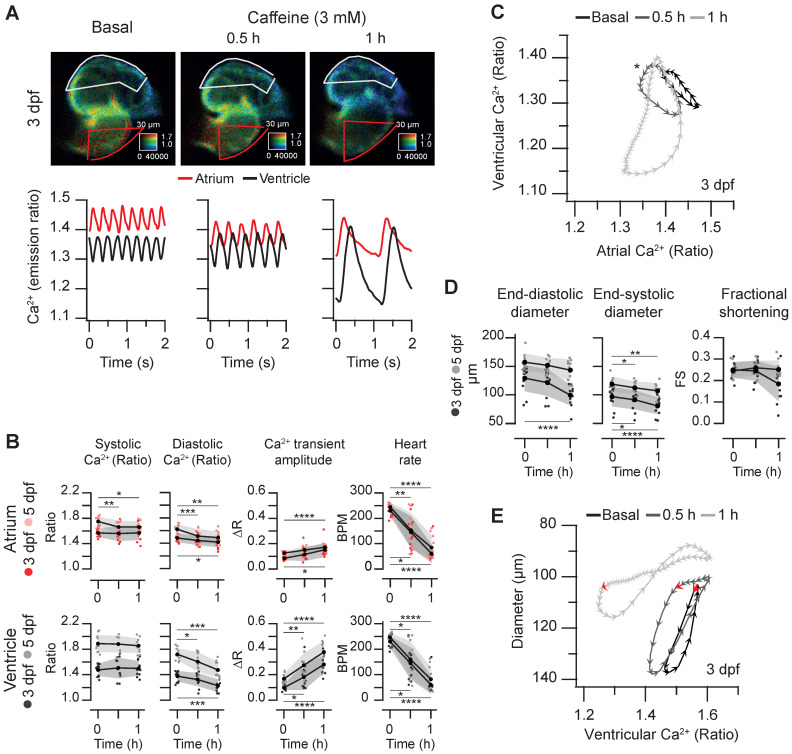
** Effect of caffeine on cardiac Ca^2+^ levels and ventricular shortening in 3 and 5 dpf zebrafish larvae.** *Tg(myl7:Twitch-4)* larvae at 3 (n = 12, N = 3) and 5 dpf (n = 11, N = 4) were treated with 3 mM caffeine. **(A)** Emission ratio images of ventricular diastoles of a representative larva before (basal) and after 0.5 and 1 h incubation with caffeine (representative experiment). The traces show the corresponding atrial and ventricular Ca^2+^ levels (emission ratio). **(B)** Systolic and diastolic Ca^2+^ levels, Ca^2+^ transient amplitude and heart rate before and after 0.5 and 1 h treatment with caffeine **(C)** Diagram of the ventricular *vs*. atrial Ca^2+^ levels (one cardiac cycle) from the larva in A (3 dpf). **(D)** Ventricular diameters and FS before and after 0.5 and 1 h incubation with caffeine. **(E)** Diagram of the ventricular diameter *vs*. ventricular Ca^2+^ level (one cardiac cycle) of a representative 3 dpf larva before and after 0.5 and 1 h incubation with caffeine. The red arrows mark the start of the atrial systole and ventricular filling. Data in (B) and (D) are shown as the mean (black line) ± SD (gray stripe). Statistical analysis was performed as indicated in Supplementary data 1 (* p < 0.05; ** p < 0.01; *** p < 0.001, **** p < 0.0001).

Caffeine is structurally related to adenosine and acts as an A_1_ and A_2A_ adenosine receptor antagonist and as a non-selective competitive inhibitor of phosphodiesterases, raising intracellular cAMP concentration. Although it has been used in isolated cardiomyocytes to release Ca^2+^ from the sarcoplasmic reticulum 1, it may act on several targets and organs in zebrafish larvae, including the central nervous system. We tested the effect of caffeine on the spontaneous cardiac Ca^2+^ transients and heart contractions at 3 and 5 dpf. Caffeine at 0.1 mM showed minor effects and at 1 mM it induced mild bradycardia after 1 hr (Figure S6 and Supplementary Data 1). At 3 mM, there was a marked time-dependent decrease of the HR and a large decrease of the diastolic Ca^2+^ levels mostly in the ventricle, whereas systolic Ca^2+^ levels were less affected (Figure 7A-B and Movie S4). The amplitude of the Ca^2+^ transients increased, particularly in the ventricle (Figure 7B). The changes in HR and amplitude were patent in the diagram of ventricular *vs*. atrial Ca^2+^ (Figure 7C). Before caffeine, as in control larvae (Figure 3B-D) atrial and ventricular Ca^2+^ were out of phase. At 30 min of treatment, the phase of simultaneous decrease of Ca^2+^ in atrium and ventricle was prolonged due to the lower HR, widening the loop (asterisk in Figure 7C). After one hour incubation, the duration of a cardiac cycle increased even more and the rise in atrial Ca^2+^ was followed shortly by a rise in ventricular Ca^2+^. Finally, both atrial and ventricular Ca^2+^ levels decreased in parallel in a prolonged relaxation phase (Figure 7A and C; Movie S4).

Regarding the effects of caffeine on heart contraction, the ventricular diameters decreased, but no significant change in the FS was observed (Figure 7D). In keeping with the slower HR, the contraction phase and, particularly, the relaxation phase slowed down (Movie S4); in the representative larva of Figure 7E, the systole before caffeine addition lasted 120 ms (6 arrowheads), while after 1 hour caffeine it lasted 260 ms. During the decay of the Ca^2+^ transient, the ventricular diameter did not increase much until the onset of atrial contraction (marked by red arrows in Figure 7E), which gave rise to ventricular filling in diastole. End-diastolic volume in zebrafish is known to depend largely on atrial contraction 13.

**Figure 8:**
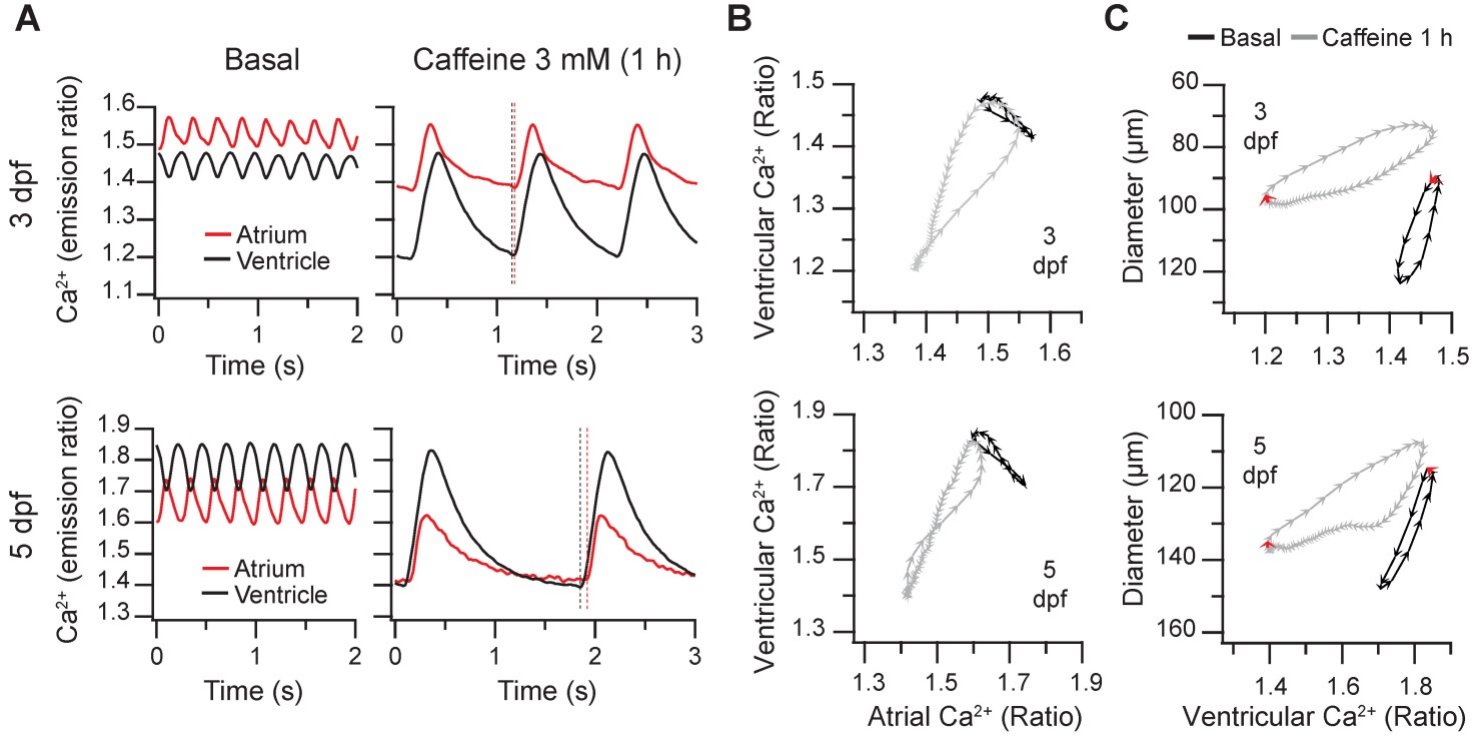
** Altered excitation in 3 and 5 dpf zebrafish larvae caused by caffeine.** Some *Tg(myl7:Twitch-4)* larvae treated with 3 mM caffeine showed an altered pattern of atrial and ventricular Ca^2+^ transients. **(A)** Atrial and ventricular Ca^2+^ levels (emission ratio) before (basal) and after 1 h incubation with 3 mM caffeine in 3 and 5 dpf larvae (representative experiments). The vertical dotted lines show that ventricular Ca^2+^ increased before atrial Ca^2+^ in the 5 dpf larva. **(B)** Diagrams of the ventricular *vs*. atrial Ca^2+^ levels (one cardiac cycle) of the larvae in A before (basal) and after 1 h incubation with caffeine. **(C)** Diagrams of the ventricular diameter *vs*. ventricular Ca^2+^ level (one cardiac cycle) of the same larvae. The red arrows indicate the start of the atrial systole. Each arrowhead in B and C corresponds to one image: they are separated from each other by 20 ms.

In 4 larvae out of 12 (3 dpf) and 2 out of 11 (5 dpf) treated with 3 mM caffeine, the normal atrioventricular direction of excitation was altered (Figure 8A and Movie S5). The Ca^2+^ rise in atrium and ventricle was simultaneous (at 3 dpf, both systoles were in phase), or even it started earlier in the ventricle (at 5 dpf, vertical dashed lines in Figure 8A and Movie S5) suggesting failure of the sinoatrial pacemaker and an escape rhythm. Therefore, the slope of the atrial *vs*. ventricular Ca^2+^ loops changed by about 90º showing that Ca^2+^ rise was in phase in both chambers. In these larvae atrial contraction (red arrows in Figure 8C) occurred together with ventricular Ca^2+^ rise and shortening and did not contribute to ventricular filling. This kind of analysis will be useful to identify altered patterns caused by drugs or mutations.

## Discussion

Detailed electrophysiological and mechanobiological studies have been reported in cardiomyocytes mostly derived from rodents and from human induced pluripotent stem cells 46. For videomicroscopy studies in cardiomyocytes, a goal has been to automate image analysis, generating readouts like HR, beat duration and amplitude, beat-to-beat variation, and sarcomere contraction and relaxation parameters 47, 48. In reports of cardiac performance in zebrafish, several methods based on bright-field 49-51 and wide-field or light-sheet fluorescence microscopy 52, 53 have provided estimates of mechanical and contractile function of the heart, such as the HR, end-systolic and end-diastolic volumes, ejection fraction, stroke volume and cardiac output. Moreover, zebrafish lines of fluorescent biosensors have been generated to provide a readout of voltage and/or Ca^2+^ levels in the heart 31, 54, 55. In this study, we obtained simultaneously mechanical performance endpoints and Ca^2+^ dynamics parameters in zebrafish larvae stably expressing the GECI Twitch-4.

The response kinetics of a biosensor is essential to analyze dynamic Ca^2+^ concentration changes. The kinetics of GECIs is slower than that of synthetic Ca^2+^ indicators 38, 56-58. In the Twitch series, an expected inverse relationship was seen between Ca^2+^ affinity and kinetics. Thus, biosensors with decreasing Ca^2+^ affinity (Twitch-2B, -3, -4 and -5) showed decay time constants of 2.8, 1.5, 0.5 and 0.16 s, respectively 45. Hence our choice of Twitch-4, with relatively fast kinetics and low Ca^2+^ affinity (*Kd* of 2.8 µM) to minimize kinetics dampening.

The *in vitro* kinetics is difficult to compare between Ca^2+^ biosensors because measurements in the literature are done in different experimental conditions, like ionic strength and temperature. In addition, the cell milieu differs considerably from the *in vitro* conditions. The *k*on of Twitch-4 can be calculated from the published *Kd* and *k*off. It is 7 × 10^5^ M^-1^ s^-1^, compared to ~10^7^ M^-1^ s^-1^ for various GCaMP variants that are considered fast GECIs. The *k*off of Twitch-4 and GCaMPs is more similar (2 s^-1^ and 3-4 s^-1^, respectively) 56. In view of these *in vitro* parameters, GCaMP fluorescence should report the start of the Ca^2+^ transient more accurately than Twitch-4 emission ratio. Yet, we show here that Twitch-4 was able to track systolic Ca^2+^ transients like GCaMP, a widely used biosensor in zebrafish heart studies. Figure 2C shows that the rise and decay time of Twich-4 in atrium and ventricle (in beating hearts) were identical to those recorded for GCaMP (in MO-arrested hearts). Since our experiments were done at 28 °C, it is likely that Twitch-4 and GCaMP had faster kinetics than *in vitro* at a lower temperature.

The *Kd* of Twitch-4 determined *in vitro* (2.8 µM) 38 was well suited to detect both rises and decreases of Ca^2+^ levels (Figures 5 and 6). When intensiometric Ca^2+^ biosensors are employed, fluorescence along time is usually normalized to the diastolic or the lowest fluorescence of the recording (F/F_0_), so no inference can be made on the diastolic levels across experiments or larvae. It has been argued that this is a reason why relatively little is known about the regulation of diastolic Ca^2+^ levels in the heart 9. In contrast, with Twitch-4, differences in Ca^2+^ levels were seen not only during the cardiac cycle or after addition of drugs, but also among larvae. The ratio changes were suggestive of different Ca^2+^ levels at 3 and 5 dpf, and we confirmed this contention with an independent method based on GFP-aequorin bioluminescence 43.

Within the ample toolbox of GECIs 30, 45, 59, ratiometric biosensors have distinct advantages over intensiometric ones for imaging a motile organ like the heart. As we showed in an earlier study 36 and here in Figure 2, ratiometric imaging with FRET biosensors largely corrected the motion artifacts and allowed imaging Ca^2+^ changes in beating hearts. These results agree with Tsutsui *et al*. 55, who reported voltage mapping with a ratiometric genetically encoded biosensor in beating hearts of zebrafish larvae. The positively correlated fluctuations of the two fluorescence channels due to focusing and defocusing and motion of the heart cancelled well in the ratio, whereas the reciprocal components due to the voltage-dependent FRET were amplified. An alternative to FRET biosensors is to co-express an intensiometric GECI like GCaMP with a static reference (DsRed): their emission ratio cancels out the motion and defocusing artifacts, leaving only the Ca^2+^-dependent changes 60. A technical detail allowed us to quantify Ca^2+^ with static ROIs, in spite of the lateral movement of the atrium and ventricle in the focal plane and to a lesser extent in the z axis. The contribution of each pixel to the average ratio was weighted by its fluorescence intensity 34, 35, so that dim pixels had negligible influence and the ROIs did not need to match the shape of the heart chambers in each video frame.

In keeping with the contention that motion artifacts were cancelled in our study, the temporal shapes and rates of the Twitch-4 ratio changes in beating hearts were consistent with those of hearts stopped with a myosin inhibitor or *tnnt2a* morpholino (Figure 2B, Twitch-4+PAB; Figure 2C, GCaMP+morpholino) and with those in previous reports 31, 32. Although zebrafish larvae can survive without circulation up to 5 dpf, it has been shown that preventing blood flow with *tnnt2a* morpholinos caused endothelial cell apoptosis in vascular plexus of zebrafish embryos 61. Furthermore, in a patch-clamp study it was found that mechanical load may affect the action potential duration, Ca^2+^ signaling and contractility of mouse ventricular myocytes 62, which underlines the importance of mechano-transduction in cardiomyocytes. Therefore, in our study no myosin inhibitors or morpholinos were used to stop heart beating, so the heart mechano-electrical feedback mechanisms were intact. A key aspect of this work was the automatic extraction of Ca^2+^ and contraction parameters from the Twitch-4 fluorescence images with a custom written analysis program. We tracked contraction of the ventricle alongside with Ca^2+^ in each experiment. Since the biochemical trigger (Ca^2+^) and its mechanical outcome were gathered in real time at 50 or 100 Hz (fps), analysis of their phase-plane trajectories (Lissajous diagrams) provided a visual snapshot of the changes occurring during development (Figure 4D) or as a result of drug addition (Figures 5E, 6D, 7E and 8C). The correlation of Ca^2+^ with contraction has been studied in isolated cardiomyocytes 19, 63, 64 but, to our knowledge, this is the first demonstration *in vivo*.

A limitation in our study and similar reports in zebrafish is that the internal concentration in larvae of the drugs added to the bath is generally unknown. Often, higher concentrations must be used to see effects, compared to *in vitro* studies. In our experiments the layer of agarose over the larvae was 1.9 mm thick and over this distance diffusion is slow. It is possible to remove part of the agarose to increase drug access but, as this must be done manually, it may introduce a source of variability between larvae. In addition, drugs must cross the skin to reach internal organs. In one report, Parker *et al.* measured the concentration in larvae by liquid chromatography with tandem mass spectrometry and compared it to the bath concentration of various compounds 50. They found 0.004-fold internal *vs*. external concentration for some drugs (adrenaline and ouabain), 0.07 for theophylline, 1- to 2-fold for verapamil and terfenadine, and yet 20-fold for cisapride and haloperidol, highlighting the importance of bioanalysis to interpret results, especially if effects are not observed 50. To follow the effects of slow-acting drugs, we repeated measurements every 5 to 15 min (i.e. Figure 6B). An alternative method by cardioluminescence with GFP-aequorin allows non-stop imaging for extended periods (hours) 43.

Caffeine is known to increase the Ca^2+^ leak of the SR by sensitizing the RyR to the luminal Ca^2+^ level 1, but in our experiments *in vivo*, it may act on several targets. The major effects observed with caffeine were a marked decrease in HR, reduced diastolic Ca^2+^ levels and an increase in the size of the Ca^2+^ transients (Figure 7). These effects, observed 1 hr after caffeine addition, are compatible with the regulation of diastolic Ca^2+^ by the HR and with the maintenance of flux balance at steady state 1, 9. At the spontaneous HR of zebrafish larvae of about 4 Hz, diastolic Ca^2+^ levels were elevated, since Ca^2+^ transients did not have enough time to decay before the next Ca^2+^ transient occurred. Concurrently, the amplitude of the Ca^2+^ transient was small, likely due to a decrease in the LTCC current. Thus, Zhang *et al.* reported in zebrafish cardiomyocytes that I_Ca,L_ decreased with shorter stimulation intervals (from 2 to 0.33 s), due to inactivation of I_Ca,L_ by Ca^2+^, since it was associated with an increase in the diastolic Ca^2+^ levels (see Figure 5 in 19). In our results, the HR after caffeine fell to less than 1 Hz, the diastolic Ca^2+^ levels decreased, likely approaching resting Ca^2+^ (the level observed in the absence of stimulation), and the amplitude of the Ca^2+^ transients increased (Figure 7 and Movie S4). We have also seen large ventricular Ca^2+^ transients associated with low HR provoked by drugs like dofetilide and astemizole (manuscript in preparation).

Caffeine *in vivo* has a complex pharmacology. In the literature there are reports of bradycardia induced by caffeine in humans 65. Although the mechanism is unclear, there is evidence that inhibition of adenosine receptors by caffeine leads to high blood pressure, which induces an autonomous reflex and bradycardia. In addition, previous reports in zebrafish of 2 and 3 dpf showed a dose-dependent decrease in heart rate by caffeine 66. As discussed above, the embryo/bath concentration of theophylline reported by Parker et al. was 0.07 50. Caffeine and theophylline have very similar structures, only differing in a methyl group; their XLogP values are 1.55 and 1.68, respectively. If we assume that they would have similar embryo/bath ratios, 1 mM caffeine in the bath should result in an embryo concentration of about 70 µM, consistent with an effect on adenosine receptors at 10-100 µM (pKi 4 to 5) 67.

Automaticity in cardiac muscle is believed to be regulated by voltage-gated calcium channels, in addition to HCN channels (I_f_ current) 68. An additional mechanism for pacemaker depolarization involves the rhythmic Ca^2+^ release from SR that triggers an inward current through the NCX1. The loss of this current specifically in the atrium in KO mice abolished the sinus pacemaker activity and caused an idioventricular escape rhythm, although atrial tissue remained excitable 69. In our study, in some larvae treated with caffeine, an escape rhythm appeared (Figure 8). Leak of Ca^2+^ from the SR of sinoatrial cells in these larvae by caffeine could eliminate the local Ca^2+^ release, the ensuing inward current through the NCX and sinus pacemaker activity. The characterization of caffeine effects on Ca^2+^ levels (Figure 7 and 8) underscores the potential of this method to investigate drug-induced and familial arrhythmias and their functional consequences in zebrafish larvae.

## Conclusions

The *Tg(myl7:Twitch-4)* line offers a physiological, non-invasive approach to image Ca^2+^ in the heart of zebrafish embryos and larvae, allowing estimation of systolic and diastolic Ca^2+^ levels, while keeping intact the electromechanical and paracrine regulatory mechanisms. Measurement of changes in contractility is critical for appraisal of cardiac function and this method affords simultaneous registration and correlation of Ca^2+^ and contraction. In the last years several models of heart failure and cardiomyopathy have been reported in zebrafish, and gene-editing by CRISPR-Cas9 allows to humanize zebrafish models by introducing the normal or mutated human orthologue genes 18, 22. The current line expressing Twitch-4 will be a valuable tool to characterize their pathophysiology, whether Ca^2+^ fluxes are involved and their impact on heart contractility. In addition, cardiotoxicity studies and screening for new drugs could be performed. The applicability of this approach for drug screening is currently limited by the handling and positioning of embryos in agarose in multi-well plates. Automation of this step 26, 27 would allow for the sampling of about 60 larvae per hour. Integration of *in vivo* data from zebrafish models with the extensive literature in cardiomyocytes and isolated hearts will likely contribute to unravel the mechanisms of heart disease progression and to identify therapeutical targets.

## Supplementary Material

Supplementary figures and movie legends.

Supplementary information.

Supplementary movie 1.

Supplementary movie 2.

Supplementary movie 3.

Supplementary movie 4.

Supplementary movie 5.

## Abbreviations

At: atrium
bpm: beats per minute
dpf: days post-fertilization
ECG: electrocardiogram
FAC: fractional area change
fps: frames per second
FRET: Förster resonance energy transfer
FS: fractional shortening
GECI: genetically encoded Ca^2+^ indicator
HCN channels: Hyperpolarization-activated cyclic nucleotide-gated channels
hpf: hours post-fertilization
HR: heart rate
I_Ca,L_: L-type Ca^2+^ current
LTCC: L-type voltage-dependent Ca^2+^ channel
NCX: sodium-calcium exchanger
PAB: para-amino blebbistatin
ROI: region of interest
RyR: ryanodine receptor
SR: sarcoplasmic reticulum
Vt: ventricle
