# Supplementary material for: Simultaneous imaging of calcium and contraction in the beating heart of zebrafish larvae
Source: Theranostics. 2022 Jan 1;12(3):1012–29. doi: 10.7150/thno.64734 (PMC8771564; doi:10.7150/thno.64734)
Supplement: Supplementary file 1 — Supplementary figures and movie legends. [file thnov12p1012s1.pdf]

Simultaneous imaging of calcium and contraction in the beating heart of zebrafish larvae

Jussep Salgado-Almario[1], Manuel Vicente[1], Yillcer Molina[1], Antonio Martinez-Sielva[1], Pierre Vincent[2*], Beatriz Domingo[1*] and Juan Llopis[1*]

[1]Physiology and Cell Dynamics, Centro Regional de Investigaciones Biomédicas (CRIB) and Facultad de Medicina de Albacete, Universidad de Castilla-La Mancha, C/Almansa 14, 02006 Albacete, Spain

[2]Sorbonne Université, CNRS, Biological Adaptation and Ageing, UMR 8256, F-75005 Paris, France

Supplementary figures

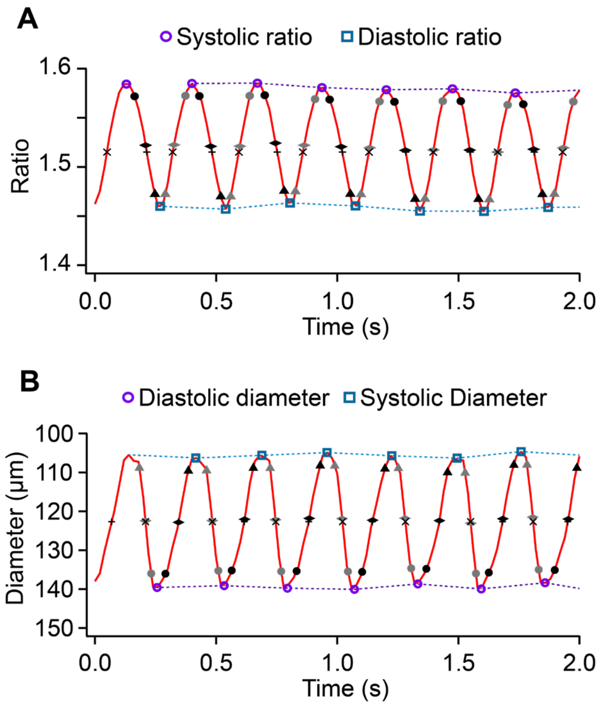

Figure S1. Automatic detection of points of interest on the time-course of Twitch-4 emission ratio and ventricular diameter. The edge detection functions in the Igor Pro software (WaveMetrics) were implemented in the custom-made analysis module “Pulse” in the “Ratioscope” analysis software (see Image processing and data analysis in Methods). **(A)** Detection of points of interest on the time-course of Ca^{2+} levels (trace obtained from Twitch-4 fluorescence emission ratio). **(B)** Detection of points of interest on the time-course of ventricular diameter (trace obtained from Twitch-4 EYFP emission, FRET channel).

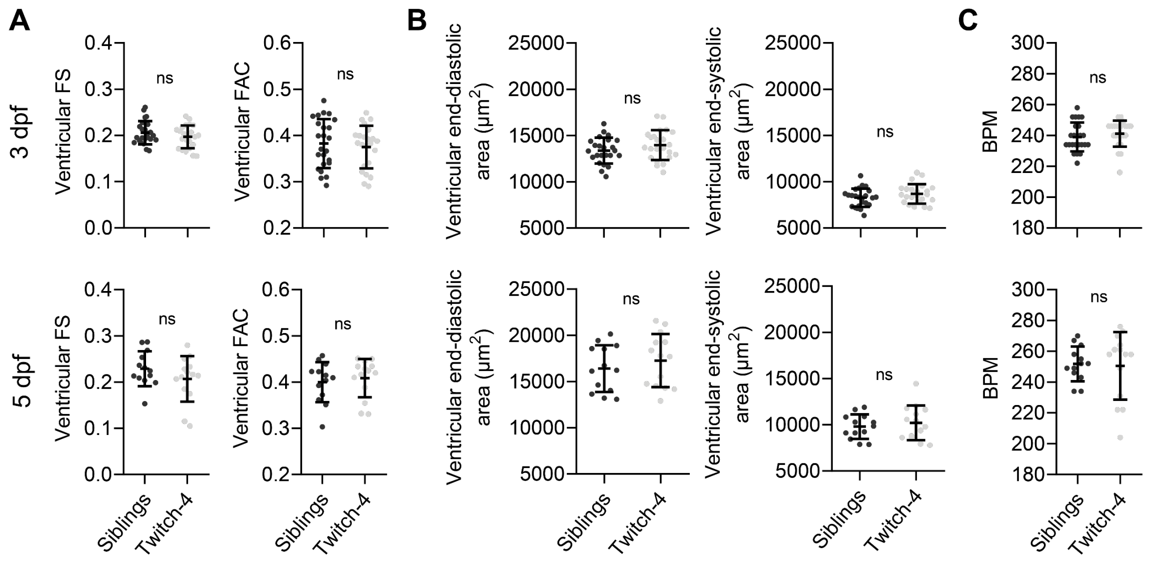

Figure S2. Effects of the expression of Twitch-4 on the force of contraction, the size of the embryonic zebrafish heart and the heart rate. Cardiac parameters were estimated by transmitted light in *Tg(myl7:Twitch-4)* larvae (n = 23, N = 3 at 3 dpf and n = 14, N = 3 at 5 dpf) and their non-expressing siblings (n = 24, N = 3 at 3 dpf and n = 13, N = 3 at 5 dpf). **(A)** Ventricular FS and ventricular FAC. **(B)** Ventricular end-diastolic and end-systolic areas. **(C)** Heart rate in bpm. Data are shown as the mean ± SD. Statistical analysis was performed as indicated in Supplementary data 1.

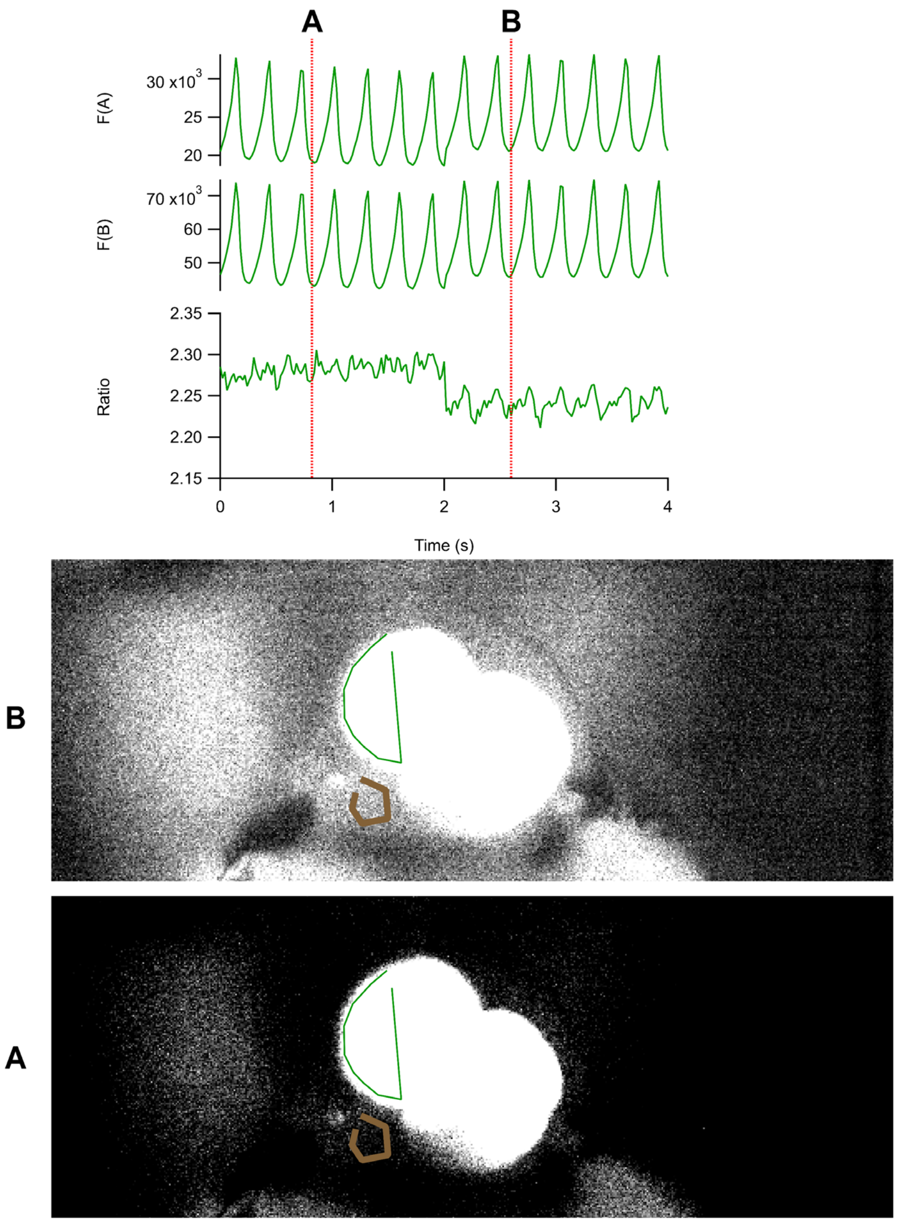

Figure S3. The ratio fluctuations in synch with contraction in the *Tg(myl7:ECFP-16aa-EYFP)* line are due to yolk autofluorescence under the heart. The beating heart of a *Tg(myl7:ECFP-16aa-EYFP)* larva at 3 dpf was imaged as in Figure 2A-B. The green ROI was placed over the ventricle and the brown ROI next to it was used to measure background fluorescence.

In our image analysis protocol, we did not subtract background fluorescence of the biological sample. In this experiment, from time 0 to 2 s the ECFP (F(A)) and FRET (F(B)) fluorescence channels and the emission ratio were recalculated by subtracting the average fluorescence measured over a ROI next to the ventricle (labelled in brown in the images) to the raw images. From time 2 to 4 s the fluorescence levels and ratio without background subtraction are shown. Image A shows the background subtracted image (corresponding to point A in the top traces) in the acceptor channel. Image B shows the raw un-subtracted image (point B in

the top traces). The images have been scaled to show the autofluorescence surrounding the heart (which looks saturated). The ratio trace from 2 to 4 s (point B) shows periodic ratio fluctuations in synch with the cardiac cycle (as in Figure 2B). The result of subtracting the background measured in a ROI next to the ventricle was that the periodic fluctuations mostly disappeared (ratio trace from 0 to 2 s; point A). Therefore, most of the fluctuations seen in ECFP-16aa-EYFP (Figure 2B) are due to the autofluorescence under the heart caused by the yolk.

Image B illustrates the spatial heterogeneity of background fluorescence. It changed from embryo to embryo, depending on the size of the yolk sac and the orientation of the heart. It affected more the atrium (Figure 2B) since Twitch-4 fluorescence was dimmer than in the ventricle. In addition, larvae at 3 dpf showed more autofluorescence than at 5 dpf, as the yolk sac was larger. Therefore, we did not attempt to subtract autofluorescence in Twitch-4 imaging since such objective measurement is not feasible. Instead, we used the intensity weighted method to calculate the average ratio over a ROI [34,35], which reduces the contribution of pixels with autofluorescence or out-of-focus fluorescence out of the heart. We find this method more objective, though not perfect, hence the residual oscillation of the ratio. Of note, yolk and embryo autofluorescence will likely affect measurements done with any fluorescent biosensor. Importantly, the motion artifact was small compared to the calcium signal: the comparisons in Figure 2B-C between stopped and beating hearts show that it was of minor concern since the specific Twitch-4 fluorescence was dominant. See also the small difference in the fluorescence intensity in the top traces of Figure S3 with background subtraction (0 to 2 s) and without it (2 to 4 s).

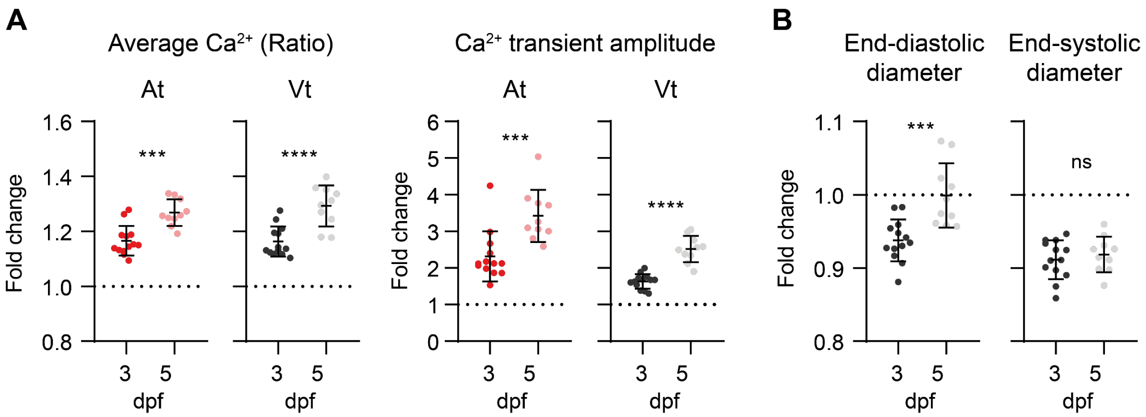

Figure S4: Effect of the L-type Ca^{2+} channel activator Bay K8644 on the average Ca^{2+} levels, Ca^{2+} transient amplitude and ventricular diameter. *Tg(myl7:Twitch-4)* larvae at 3 (n = 13, N = 3) and 5 dpf (n = 10, N = 3) were treated with 100 µM Bay K8644 for 10 min. **(A)** Fold change of the average Ca^{2+} levels and amplitude of Ca^{2+} transients over their basal values in the atrium (At) and ventricle (Vt). **(B)** Fold change of the ventricular end-diastolic and end-systolic diameters over their basal values. All data are shown as mean ± SD. A two-tailed unpaired Student's t-test was used except for the atrial Ca^{2+} transient amplitude, where a Mann-Whitney test was used (*** $p < 0.001$, **** $p < 0.0001$).

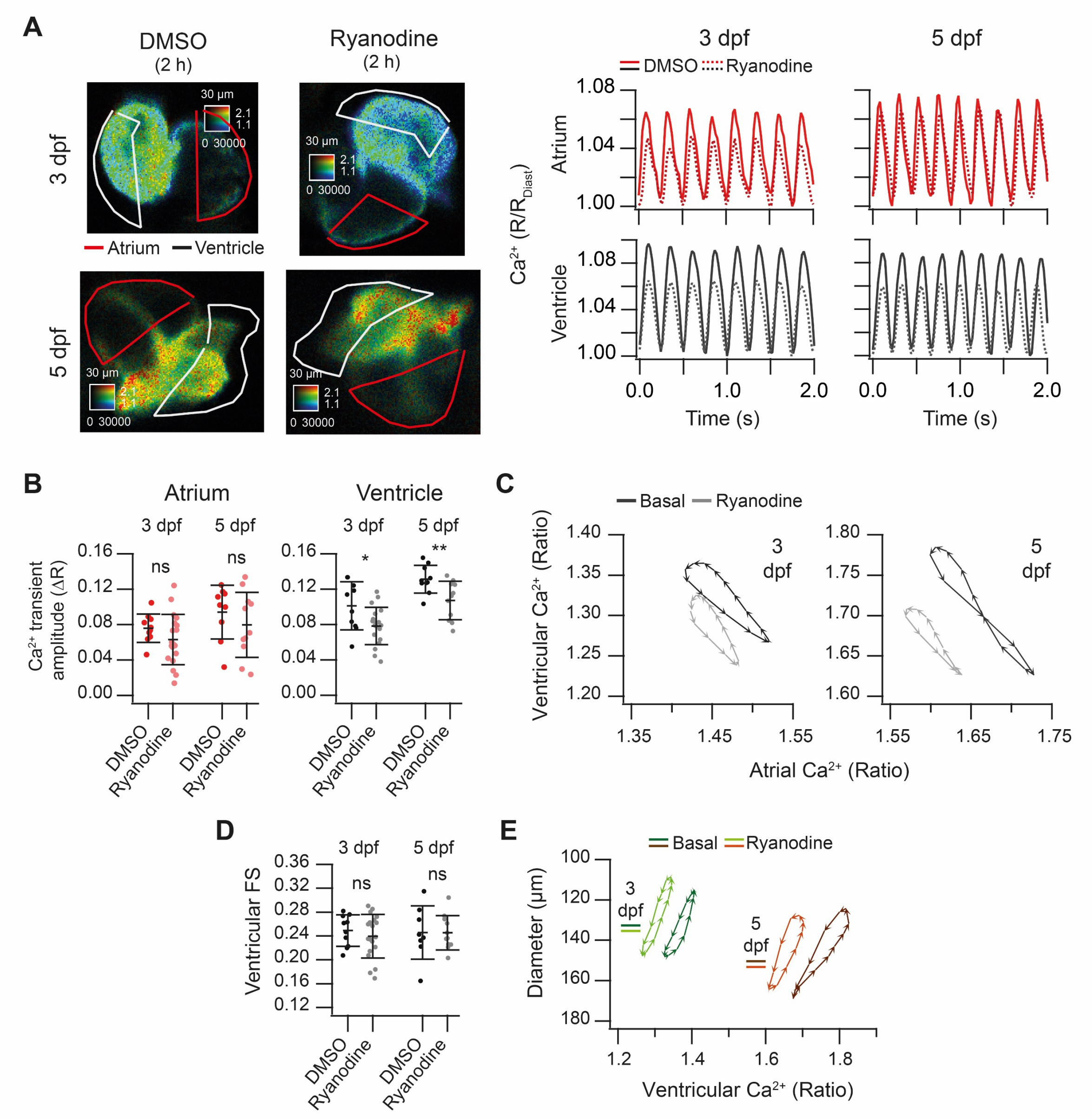

Figure S5: Effect of ryanodine on cardiac Ca^{2+} levels and ventricular shortening in 3 and 5 dpf zebrafish larvae. *Tg(myl7:Twitch-4)* larvae at 3 and 5 dpf were treated with 100 µM ryanodine (n = 19, N = 4 at 3 dpf; n = 11, N = 3 at 5 dpf) or with 1% DMSO (n = 10, N = 4 at 3 dpf; n = 9, N = 3 at 5 dpf) for 2 h. **(A)** Emission ratio images of representative larvae in ventricular systole after incubation with DMSO or ryanodine. The traces show the atrial (red) and ventricular (black) emission ratio traces over time calculated from these larvae. **(B)** Effect of ryanodine on the Ca^{2+} transient amplitude compared to larvae treated with DMSO. **(C)** Diagrams of the ventricular vs. atrial Ca^{2+} levels (one cardiac cycle) of representative larvae before (basal) and after incubation with ryanodine. **(D)** Effect of ryanodine on the ventricular FS. **(E)** Diagrams of the ventricular diameter vs. ventricular Ca^{2+} level (one cardiac cycle) of representative larvae before (basal) and after 2 hours incubation with ryanodine. Data in B and D are shown as the mean ± SD. A two-tailed unpaired Student's t-test was used (* $p < 0.05$, ** $p < 0.01$).

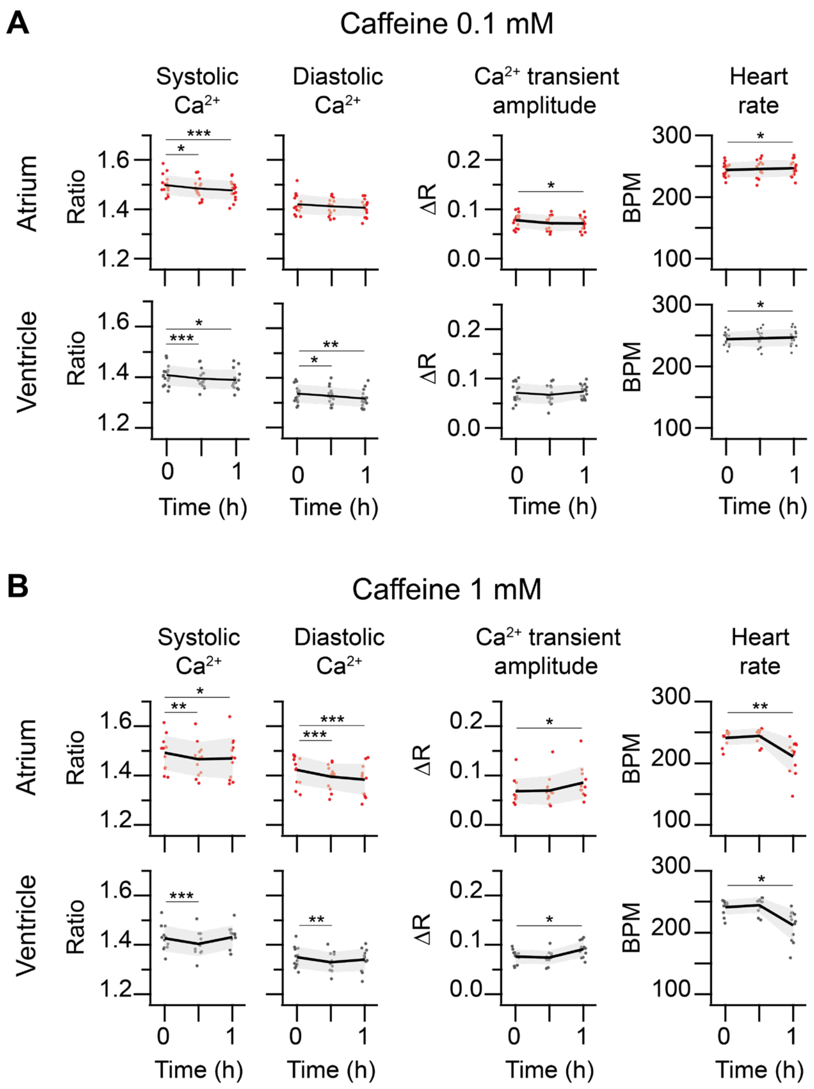

Figure S6: Effect of 0.1 mM and 1 mM caffeine on cardiac Ca^{2+} levels in 3 dpf zebrafish larvae. *Tg(myl7:Twitch-4)* larvae at 3 dpf were treated with 0.1 mM (n = 16, N = 3) **(A)** or 1 mM (n = 12, N = 3) **(B)** caffeine. Systolic and diastolic Ca^{2+} levels, Ca^{2+} transient amplitude and heart rate before and after 0.5 and 1 h incubation with caffeine in atrium and ventricle are shown. Data are shown as the mean (black line) ± SD (gray stripe). Statistical analysis was performed as indicated in Supplementary data 1 (* $p < 0.05$; ** $p < 0.01$; *** $p < 0.001$).

Supplementary data

Supplementary data 1. Details of the statistical analysis used in all figures.

Supplementary movies

Movie S1: Emission ratio of beating hearts in 3 and 5 dpf *Tg(myl7:Twitch-4)* larvae.

Movie S2: Emission ratio of beating hearts in 3 and 5 dpf *Tg(myl7:Twitch-4)* larvae before (basal) and after 1 h treatment with 100 µM nifedipine.

Movie S3: Emission ratio of beating hearts in 3 and 5 dpf *Tg(myl7:Twitch-4)* larvae before (basal) and after 10 min treatment with 100 µM Bay K8644.

Movie S4: Emission ratio of a beating heart in a 3 dpf *Tg(myl7:Twitch-4)* larva before (basal) and after 0.5 and 1 h treatment with 3 mM caffeine.

Movie S5: Emission ratio of a beating heart in a 5 dpf *Tg(myl7:Twitch-4)* larva before (basal) and after 1 h treatment with 3 mM caffeine, showing altered atrioventricular excitation.
