# Supplementary figures and images for: Simultaneous imaging of calcium and contraction in the beating heart of zebrafish larvae
Source: Theranostics. 2022 Jan 1;12(3):1012–29. doi: 10.7150/thno.64734 (PMC8771564; doi:10.7150/thno.64734)

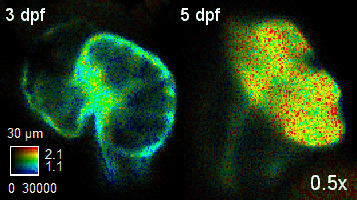

Supplement: Supplementary file 3 — Supplementary movie 1. [file thnov12p1012s3.gif]

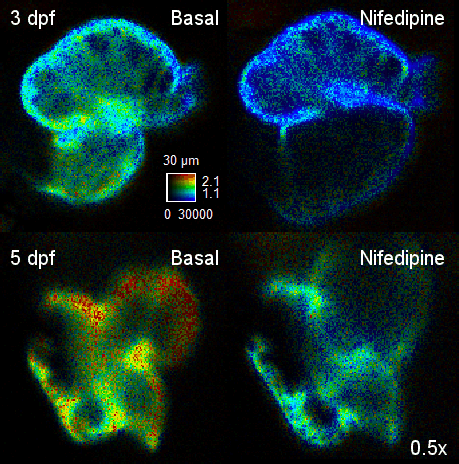

Supplement: Supplementary file 4 — Supplementary movie 2. [file thnov12p1012s4.gif]

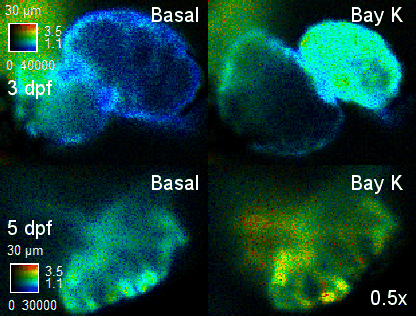

Supplement: Supplementary file 5 — Supplementary movie 3. [file thnov12p1012s5.gif]

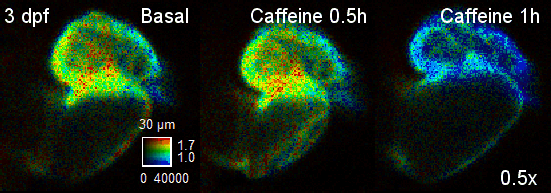

Supplement: Supplementary file 6 — Supplementary movie 4. [file thnov12p1012s6.gif]

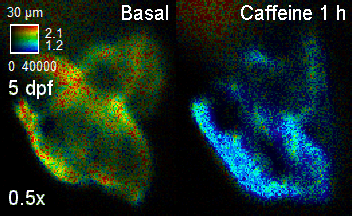

Supplement: Supplementary file 7 — Supplementary movie 5. [file thnov12p1012s7.gif]
